# Evidence for echolocation in Asian shrew moles

**DOI:** 10.1093/nsr/nwaf591

**Published:** 2025-12-26

**Authors:** Qi Liu, Qin-Yang Hua, Shui-Wang He, Lu-Ye Shi, Peng Chen, Yuan-Shuo Ma, Qin Zhang, Xue-Long Jiang, Peng Shi

**Affiliations:** State Key Laboratory of Genetic Evolution & Animal Models, Kunming Institute of Zoology, Chinese Academy of Sciences, Kunming 650223, China; State Key Laboratory of Genetic Evolution & Animal Models, Kunming Institute of Zoology, Chinese Academy of Sciences, Kunming 650223, China; Kunming College of Life Science, University of Chinese Academy of Sciences, Kunming 650223, China; State Key Laboratory of Genetic Evolution & Animal Models, Kunming Institute of Zoology, Chinese Academy of Sciences, Kunming 650223, China; State Key Laboratory of Genetic Evolution & Animal Models, Kunming Institute of Zoology, Chinese Academy of Sciences, Kunming 650223, China; State Key Laboratory of Genetic Evolution & Animal Models, Kunming Institute of Zoology, Chinese Academy of Sciences, Kunming 650223, China; State Key Laboratory of Genetic Evolution & Animal Models, Kunming Institute of Zoology, Chinese Academy of Sciences, Kunming 650223, China; State Key Laboratory of Genetic Evolution & Animal Models, Kunming Institute of Zoology, Chinese Academy of Sciences, Kunming 650223, China; State Key Laboratory of Genetic Evolution & Animal Models, Kunming Institute of Zoology, Chinese Academy of Sciences, Kunming 650223, China; State Key Laboratory of Genetic Evolution & Animal Models, Kunming Institute of Zoology, Chinese Academy of Sciences, Kunming 650223, China; School of Future Technology, University of Chinese Academy of Sciences, Beijing 101408, China; Center for Excellence in Animal Evolution and Genetics, Chinese Academy of Sciences, Kunming 650223, China

**Keywords:** echolocation, Uropsilinae, mammals

## Abstract

Echolocation is an adaptive sensory behavior that enables animals to navigate and perceive their surroundings in environments where vision is ineffective. This ability has been hypothesized to be widespread among nocturnal mammals with reduced eyesight, particularly in insectivorous moles. Here, we found that Asian shrew moles (subfamily Uropsilinae) produce ultrasonic pulses in the frequency range of 50∼100 kHz, which structurally resemble frequency-modulated echolocation calls found in other well-known echolocating mammals. Behavioral experiments further confirmed that Asian shrew moles use these ultrasonic pulses for echolocation. Analysis of the acoustic features of their ultrasonic calls, the morphology of their echolocation-related stylohyal bone, and tongue ligation experiments indicate that these Asian shrew moles produce echolocation calls using their tongues. Our findings demonstrate that Asian shrew moles represent a new echolocating mammalian lineage, indicating that echolocation independently occurred at least six times in mammals and underscoring the likelihood that the species diversity of echolocating mammals has been significantly underestimated.

## INTRODUCTION

Among numerous cases under convergent evolution [1–6], echolocation is a prominent example, having independently evolved in multiple lineages of vertebrates, such as birds [7], bats, whales, shrews [8], tenrecs [9], and soft-furred tree mice [10]. The repeated evolution of this trait supports the hypothesis that echolocation could potentially be widespread among mammals, especially since many mammalian species are nocturnal and possess reduced vision such as insectivorous moles (family Talpidae) (Griffin 1958). Most moles have poor eyesight and are highly adapted for a subterranean, burrowing lifestyle, typically lacking external ears [11]. As a result, it is unlikely that these burrowing moles use echolocation [12]. However, Asian shrew moles (*Uropsilus* spp.) differ notably from typical moles: they resemble shrews, are less specialized for burrowing, and lead a nocturnal, ambulatory lifestyle with prominent external ears [13]. This suggests that, unlike true burrowing moles, Asian shrew moles may possess adaptations more compatible with echolocation.

## RESULTS

### Vocalization features of Asian shrew moles

**Figure 1.**
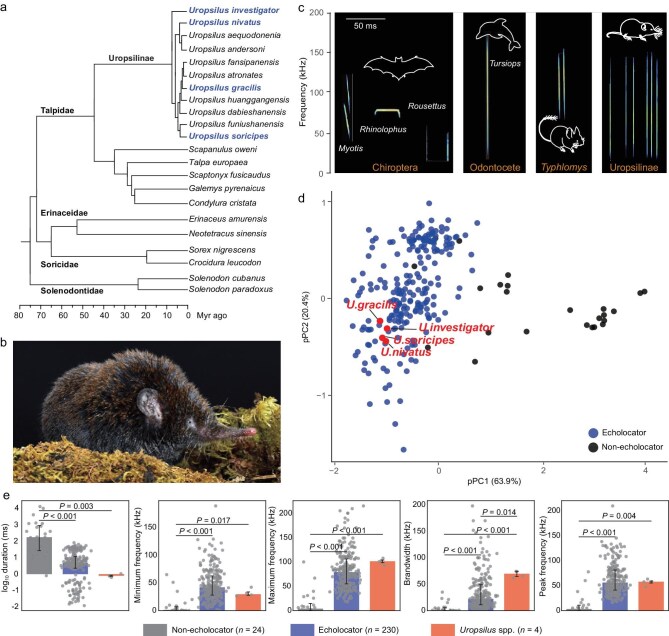
Comparisons of acoustic characteristics between Asian shrew moles and other mammals. (a) Time-calibrated species tree of the family Talpidae inferred from DNA sequence data, showing 11 recognized species of Asian shrew moles. The tree represents a consensus of phylogenetic relationships and divergence time estimations from Refs [30–32]. Four representative Asian shrew mole species examined in this study are highlighted in blue. (b) Morphology of an adult gracile shrew mole (*U. gracilis*). (c) Representative ultrasonic pulses produced by different echolocating mammals. (d) Phylogenetic principal component analysis (pPCA) of key acoustic variables (peak frequency, maximum frequency, minimum frequency, bandwidth, and pulse duration), showing the distribution of echolocating and non-echolocating mammals on phylogenetic principal component axes (pPC1 vs pPC2). Positions of Asian shrew moles are indicated by red circles. (e) Boxplot comparisons of USV acoustic variables (peak frequency, maximum frequency, minimum frequency, bandwidth, and pulse duration) among Asian shrew moles, echolocating mammals, and non-echolocating mammals. In the boxplot, the lower and upper edges of a box represent the 25% (q1) and 75% quartiles (q3), respectively. The horizontal line within a box indicates the median (md). The whiskers extend to the most extreme values within inner fences, md ± 1.5 (q3–q1). *P* values are from Mann–Whitney U tests.

To test whether Asian shrew moles possess the ability to echolocate, we first examined whether they produce regular ultrasonic vocalizations (USVs), as is typical of known echolocating mammals. Asian shrew moles represent the only extant group of the subfamily Uropsilinae, which diverged from other talpid subfamilies at the onset of the Eocene and has since diversified into 11 recognized species (Fig. 1a and b) [14]. We recorded the vocalizations of four *Uropsilus* species, including the gracile shrew mole (*U. gracilis*), the inquisitive shrew mole (*U. investigator*), the snow mountain shrew mole (*U. nivatus*), and the Chinese shrew mole (*U. soricipes*), while they moved freely in the dark. All four species emitted short, broadband USVs consisting of various pulse groups, including singles, dyads, and triads (Fig. 1c; Fig. S1). The acoustic characteristics of these USVs were highly similar among species, with a pulse duration of ∼0.8 ms, a frequency range of 26 to 107 kHz, a bandwidth of ∼69 kHz, and a peak frequency of ∼56 kHz (Table S1). To compare these acoustic features with other mammals, we performed a phylogenetic principal component analysis (pPCA) that included data from 230 echolocating and 24 non-echolocating mammalian species (Table S2). On the first two principal component axes—reflecting maximum/peak frequencies (pPC1, 63.9%) and minimum frequency (pPC2, 20.4%)—Asian shrew moles clustered closely with known echolocating species (Fig. 1d). Specifically, the peak, minimum, and maximum frequencies, as well as the bandwidth, in Asian shrew moles were comparable to those of established echolocators, and all were significantly higher than those in non-echolocating USV-producing mammals (*P* < 0.05; Mann–Whitney U tests), though the pulse duration in Asian shrew moles was significantly shorter (*P* = 0.003; Mann–Whitney U test; Fig. 1e). The regular emission of these USVs—acoustically similar to those produced by recognized echolocating mammals—strongly suggests that Asian shrew moles have evolved echolocation, with important biological implications.

### Evidence for echolocation in Asian shrew moles from behavioral experiments

**Figure 2.**
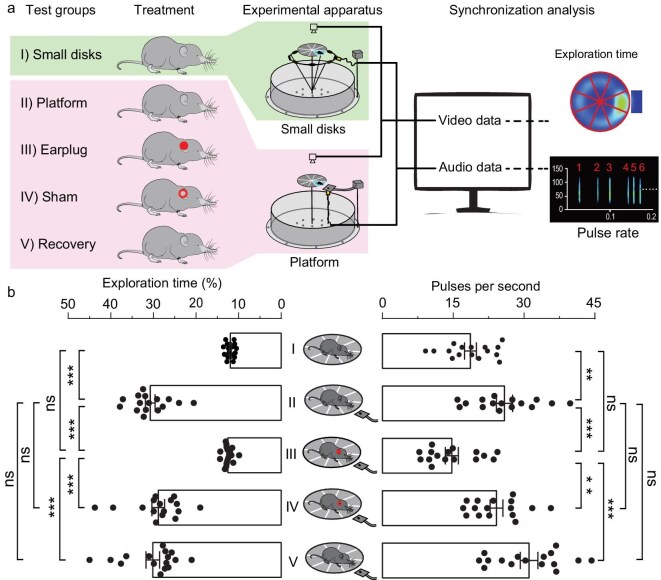
Behavioral experiments for testing echolocation in Asian shrew moles. (a) Schematic diagram of the behavioral experiments with Asian shrew moles. (b) Comparisons of the percentage of exploration time and pulse rates in the sector corresponding to the ultrasonic probe when the gracile shrew moles explored the central disc under various experimental treatments. All data were obtained from *U. gracilis*. Each dot represents the mean of the measurements of each individual (*N* = 16). The bar denotes the mean value across all individuals, and the error bars represent SEM. *P* values are from two-tailed paired Student’s *t* tests and were corrected with the Bonferroni method. **P < 0.01; ***P < 0.001; ns, not significant.

To functionally determine whether Asian shrew moles possess the ability of echolocation, we performed a series of behavioral experiments using a disc-platform apparatus, which is traditionally used to test echolocation in terrestrial mammals such as shrews [8,15], tenrecs [9], and soft-furred mice [10]. First, a disc-small disc circle was built as a control, which consisted of a raised central disc surrounded by a circle of eight small discs (Fig. 2a). The central disc was divided into eight equal sections, each corresponding to one of the small discs. A microphone was randomly placed in one of the small discs before each trial to record the sonic pulses. If an animal was placed on the central disk to explore, it was expected to spend a comparable amount of time and emit a similar number of pulses in each sector. Subsequently, the small-disc circle was replaced with a platform that was connected to a reward box by a ramp, and the position of the platform was randomly determined before each trial. If echolocation is utilized by the animal, then it should: (i) increase its exploration time and emit more sonic pulses in the sector over the platform, thus descending to the platform; (ii) lose its preference for the over-platform sector and fail to land on the platform when its ears were plugged; and (iii) regain its preference for the over platform sector when the blockages were removed or when plastic tubes that enable hearing were inserted.

When the gracile shrew moles were placed in the disc-small disc circle apparatus, they spent a similar amount of time (11.96% ± 0.24% of the total exploration time; *N* = 16 animals) and produced a similar number of ultrasonic pulses per second within each sector of the central disc in darkness (18.64 ± 1.26; *N* = 16 animals; Fig. 2b). By comparison, when in the disc-platform apparatus, the gracile shrew moles spent more time and emitted more ultrasonic pulses in the sector over the platform (*P* < 0.01; two-tailed paired Student’s *t*-tests; *N* = 16 animals; Fig. 2b), successfully detecting and dropping down to the lower platform from the central disc. However, the preference of the gracile shrew moles for the over-platform sector was lost when their ears were blocked by earplugs, performing similarly in the control tests (Fig. 2b). Following the removal or replacement of the earplugs with hollow plastic tubes, the gracile shrew moles regained the preference for the over-platform sector, spending significantly more exploration time and producing more USVs (*P* < 0.01; Fig. 2b).

To further verify the use of echolocation by gracile shrew moles, we compared the preference for the over-platform sector with its adjacent sectors. If the animals echolocate, it was expected that they would spend more time in the over-platform sector than in its adjacent sectors. Indeed, we found that the gracile shrew moles spent significantly more exploration time in the over-platform sector (30.75% ± 1.13%) than in its adjacent sectors (11.79% ± 0.61%; *P* < 0.001; Fig. S2). Furthermore, when the ears were occluded, the gracile shrew moles lost their preference for the over-platform sector in comparison with its adjacent sectors and were unable to locate the platform beneath the central disc (*P* > 0.05; Fig. S2). Upon removal of the earplugs, the gracile shrew moles regained the preference for the over-platform sector (*P* < 0.01; Fig. S2). It is noteworthy that the other three species of Asian shrew moles, *U. investigator, U. nivatus*, and *U. soricipes*, exhibited very similar performance in the aforementioned behavioral experiments (Figs S3–S5; Table S3). These findings suggest that Asian shrew moles can locate targets by emitting USVs and hearing, strongly indicating that these Asian shrew moles have echolocation ability.

### Asian shrew moles produce biosonar clicks using their tongues

**Figure 3.**
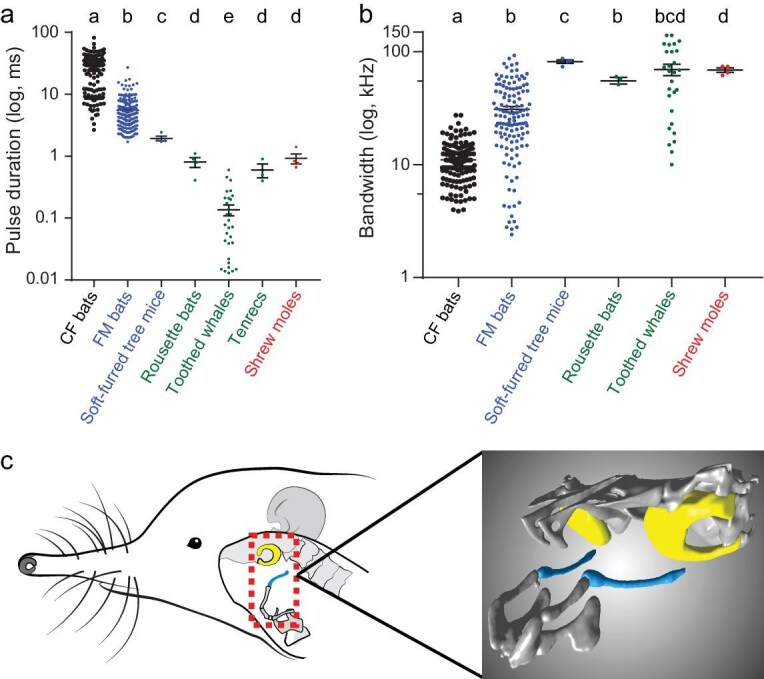
Signal features and morphology of vocal organ indicate that the Asian shrew moles generate click-like signals for echolocation. Comparisons of the duration (a) and bandwidth (b) of different types of pulses in echolocating mammals. Each dot represents a value of one echolocating species. The bar denotes the mean value across all individuals, and the error bars represent SEM. *P* values are from Mann–Whitney U tests, different letters indicate significant differences, *P* < 0.05. (c) Stylohyal and tympanic bones were separated in *U. gracilis*. Left diagram showing the spatial positions of the stylohyal and tympanic bones in a gracile shrew mole. Blue and yellow colors in the dotted box highlight stylohyal and tympanic bones, respectively. The right diagram shows the stylohyal bone separated from the tympanic bone in the gracile shrew mole.

During exploration of environments, Asian shrew moles consistently produce ultrasonic pulses of brief duration and wide bandwidth (Table S1), which were significantly shorter and broader than those of frequency-modulated (FM) and constant-frequency (CF) echolocation signals of insectivorous bats (*P* < 0.01; Mann–Whitney U tests; Fig. 3a and b). Furthermore, the duration and bandwidth of Asian shrew moles’ echolocation signals were largely comparable to the click-like echolocation signals of cave-dwelling rousette bats, toothed whales, and tenrecs (*P* > 0.05; Fig. 3a and b; Tables S1 and S2). These similar features to click-like echolocation calls suggest that the Asian shrew moles produce biosonar clicks to sense the environment [16].

Next, we sought to uncover which organ was used to generate the click-like echolocation signals in Asian shrew moles. Unlike insectivorous bats and soft-furred tree mice producing tonal echolocation signals in the larynx [10,17], the rousette bats and tenrecs generate echolocation clicks using the tongue [9,16] and toothed whales by using the phonic lips [18]. Thus, no known echolocating mammals use the larynx to produce click-like echolocation signals. To further corroborate this in the Asian shrew moles, we employed micro-computed tomography to scan the proximal articulation of the stylohyal bone with the tympanic bone, which is an anatomic feature of laryngeally echolocating mammals [17]. The results demonstrated that the proximal end of the stylohyal bone did not contact the tympanic bone in Asian shrew moles (*U.gracilis, U. nivatus*, and *U. investigator*) (Fig. 3c; Fig. S6), as in rousette bats [17].

**Figure 4.**
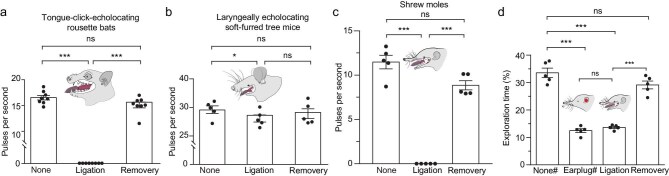
Asian shrew moles produce echolocation clicks with the tongue. Comparisons of pulse rates when echolocating mammals were tied a knot on the front of the tongue, (a) tongue-click-echolocating bats (*Rousettus leschenaultii, N* = 8), (b) laryngeally echolocating soft-furred mice (*Typhlomys daloushanensis, N* = 5), (c) gracile shrew moles (*U. gracilis, N* = 5). (d) Comparisons of the percentage of exploration time in the sector corresponding to the ultrasonic probe when the gracile shrew moles were exploring on the central disc under various experimental treatments, platform and earplug groups reference to Fig. 2, ligation group: animals tongue was tied in a knot; recovery group: knots removed. Each dot represents the mean of the measurements of each individual. The bar denotes the mean value across all individuals, and the error bars represent SEM. *P* values are from two-tailed paired Student’s *t* tests and were corrected with the Bonferroni method. *P < 0.05; ***P < 0.001; ns, not significant.

To verify that the Asian shrew moles generate click-like signals using the tongue for echolocation, we conducted a series of behavioral experiments while controlling tongue movements. The rousette bat (*Rousettus leschenaultii*) and the soft-furred tree mouse (*T. daloushanensis*) were used as the positive and negative controls, respectively. First, we ligated the tongue of the rousette bat and found that these bats failed to produce any click-like signals while flying in the dark. When the ligation was removed, the rousette bats regained the ability to generate click-like pulses (15.44 ± 0.45 per second), which was largely consistent with that before the treatment (15.12 ± 0.46) (*P* > 0.05; Fig. 4a; Table S4). By contrast, when the tongue of the soft-furred mouse was ligated, the animal still produced echolocation pulses, albeit with a reduced pulse rate (Fig. 4b; Table S4). These results strongly suggest that ligation was an effective method to assess whether the Asian shrew moles use their tongues to generate click-like signals. Upon ligation of the Asian shrew moles’ tongues, no click-like signals were produced. When the ligation was removed, the shrew moles again vocalized with a pulse rate similar to that before the treatment (*P* > 0.05; Fig. 4c; Table S4).

To determine the implications of the tongue clicks of the Asian shrew moles for echolocation, we performed behavioral experiments in the disc-platform apparatus. We found that when the tongue was ligated, the Asian shrew moles failed to produce echolocation pulses on the over-platform sector, lost their preference for that sector, and were unable to detect the platform, as they also did when their ears were blocked (*P* > 0.05; Fig. 4d; Table S5). Following removal of the ligation, the Asian shrew moles regained the capacity to produce click-like signals and significantly increased the amount of time spent exploring the over-platform sector, succeeding in landing on the platform (*P* < 0.001; Fig. 4d; Table S5). These results further confirmed that Asian shrew moles produce click-like signals for echolocation through the use of their tongues.

## DISCUSSION

Our findings provide direct evidence for echolocation in four species of Asian shrew moles. These species spanned across main evolutionary clades of Asian shrew moles, particularly *U. investigator* from the basal clade of Asian shrew moles (Fig. 1a). Consequently, it is reasonable to infer that echolocation evolved in the last common ancestors of Asian shrew moles, suggesting that all Asian shew moles have echolocation ability and that the subfamily Uropsilinae represents a new lineage of echolocating mammals. This raises the question of the origin of echolocation in Asian shrew moles. Uropsilinae is one of three subfamilies of the family Talpidae and is located at the basal position [19]. It has been noted that the majority of species in the other two subfamilies, Scalopinae and Talpinae, inhabit fully fossorial, semi-fossorial, and semi-aquatic habitats [14,19], suggesting that scalopin and talpin moles may not use echolocation. This implies that echolocation either evolved in the last common ancestor of Asian shrew moles or originated in the last common ancestor of Talpidae and was subsequently lost in the evolution of the scalopin and talpin moles. Two potential ideas can be proposed to address this question. If the fossils of the last common ancestor of the talpids could be discovered, one approach would be to examine echolocation-related features, such as cochlear width, to determine if they are consistent in echolocating shrew moles and to look for evidence of a structural reduction in scalopin and talpin moles. This comparison would indicate a single gain of echolocation in the ancestor of the talpids and a subsequent loss in scalopin and talpin moles. The other idea would be to investigate molecular convergences in genes related to echolocation between the branch of the last common ancestor of the talpids and known echolocating mammals. If a significantly higher number of convergent sites in these genes is observed, it would suggest that echolocation originated on the ancestral branch of the talpids [20,21].

Although echolocating mammals have independently evolved the ability to echolocate and show many adaptive specializations associated with sound production and hearing, a growing body of evidence indicates that they have undergone adaptively convergent amino acid replacements [10–26]. Specifically, for prestin, the motor protein in the outer hair cells of the inner ear of the mammalian cochlea, there is evidence that the number of observed convergent amino acid substitutions among echolocators significantly exceeds the chance expectation [22]. These convergent substitutions have been found to be responsible for convergent functional changes of the protein in mammalian echolocators [26]. Furthermore, besides sequence convergences in the protein-coding region, convergent evolution in the activity of *cis*-regulatory elements has also been noted among echolocating mammals [27]. It will be interesting to explore convergent evolution at the molecular level between Asian shrew moles and other echolocating mammals to decipher molecular evolutionary mechanisms underlying echolocation of Asian shrew moles in the future.

Echolocation in both shrews and Asian shrew moles suggests that the order Eulipotyphla represents the third major group of echolocating mammals, alongside the order Chiroptera and the infraorder Cetacea. Consequently, the number of mammalian orders involving echolocating species will be up to six (Table S6), accounting for ∼20% of all living mammalian orders and enabling echolocation to be an extraordinary convergent trait with the largest number of evolutionary repeatability in mammals. The discovery of echolocation in Asian shrew moles underscores the underestimates of species diversity evolving echolocation and suggests the importance of developing new high-throughput approaches that could identify new echolocating mammals. The use of machine learning methods to create predictive models of biological traits based on molecular characteristics has been demonstrated to be effective, resulting in increased throughput and reduced costs for subsequent research [28,29]. Given the significant molecular convergences among echolocating mammals, machine learning approaches are reasonable for allowing us to detect new echolocating mammals among numerous mammals with accessible high-quality genomic sequences. This systematic examination for new echolocating lineages in mammals not only empirically support Griffin’s hypothesis that echolocation is widespread among animals, but also explores the fundamental question in evolutionary genetics as to the extent to which adaptively convergent phenotypes are caused by convergent changes at the molecular sequence level.

## MATERIALS AND METHODS

### Samples of Asian shrew moles

Four species of Asian shrew moles were captured using live-traps, including *U. gracilis* (*N* = 16) from Jinfo Mountain, Chongqing City; *U. Soricipes* (*N* = 14) from Qinling Mountain, Shaanxi Province; *U. nivatus* (*N* = 11) from Yulong Mountain, Yunnan Province; and *U. investigator* (*N* = 7) from Gaoligong Mountain, Yunnan Province. These animals were individually housed in a plastic box (46 × 30 × 16 cm) in a condition-controlled room (18–25°C, 50%–70% humidity) at Kunming Institute of Zoology, Chinese Academy of Sciences. Wood shavings covered the bottom of the plastic box, and a smaller plastic box (12.7 × 7.6 × 7.6 cm) was put on one side as a nest. Asian shrew moles were fed mealworms and fruits. Water was available *ad libitum*. As Asian shrew moles are nocturnal, behavioral experiments were conducted at night. All related animal experiments were approved by the Ethics Committee of the Kunming Institute of Zoology, Chinese Academy of Sciences.

### Recording and analyzing vocalizations of Asian shrew moles

We used the Avisoft Bioacoustics CM16/CMPA microphone and the Avisoft UltraSoundGate 416H audio device (Avisoft Bioacoustics, Berlin) to perform audio recordings at a sampling rate of 250 kHz (16 bit) and frequency response of 2∼200 kHz. The recorded acoustic data were analyzed using Avisoft-SASLab Pro software v5.2 (Avisoft Bioacoustics, Berlin) in a Hamming window with a 512-point fast Fourier transform, a time-window overlap of 75%, and a threshold of 50. We used the Automatic Parameter Measurements tool (two thresholds, thresholds −10 dB, start/end −10 dB, hold time 2 ms) to obtain the acoustic parameters, including peak frequency, maximum frequency (*f*_max_), minimum frequency (*f*_min_), frequency bandwidth, pulse duration, pulse period, and pulse interval. The numbers of ultrasonic pulses were counted with the Pulse Train Analysis tool (time constant 1 ms, threshold −15 dB). The data of ultrasonic pulses from different Asian shrew mole species are shown in Table S1.

### Comparative acoustic analysis

Because species are not statistically independent due to shared ancestry, we used phylogenetic principal component analysis (pPCA) to assess functional similarity in acoustic features while accounting for phylogeny. We analyzed acoustic data from 258 mammals (230 echolocating species, 24 non-echolocating species, and 4 *Uropsilus* species) using the R package phytools [33]. Acoustic parameters were log10-transformed prior to PCA to normalize distributions and reduce the influence of extreme values. The phylogeny for the 258 species was obtained from TimeTree [34]. To compare groups, we conducted pairwise Mann–Whitney U tests on each of the five acoustic traits among echolocating species, non-echolocating species, and *Uropsilus*.

### Behavioral experiments for echolocation in Asian shrew moles

To determine whether Asian shrew moles echolocate, a disc-platform apparatus which mainly consists of two components [10]: a disc-small disc circle and a disc-platform (Fig. 2a) was used. The small-disc circle is a 40 cm diameter loop with equally arranged eight 3.6 cm diameter discs, which was used as a control. The center disc was equally divided into eight sectors, with each sector corresponding to a reward box containing freshly killed mealworms. In principle, if an animal echolocates when placed on the central disc, it should be able to use sonic signals to find the platform located some distance below the disc and jump down onto the platform without the use of tactile, olfactory, or visual information. To eliminate odors, the center disc and platform were covered with filter paper that was replaced before every trial. The ramp and reward box were rinsed with distilled water, then with 100% ethyl alcohol, and finally with distilled water before every trial. The microphone was wiped with cotton dipped in 100% ethyl alcohol and distilled water.

We first placed the small-disc ring 7 cm vertically below the center disc to conduct control tests. Then the small-disc ring was replaced with a platform that was also placed 7 cm below one of the eight sectors. The animals on the center disc cannot lean over the edge of the disc to touch the small-disc ring or the platform with their noses. According to the protocol previously described [10], we performed the behavioral experiments under various treatments in the following order: (a) the animals were placed on the disc-small disc ring apparatus without any treatment; (b) the animals were placed on the disc-platform apparatus without any treatment; (c) the animals whose ears were plugged with expandable foam were placed in the disc-platform apparatus; (d) the animals with shams (plastic tubes) in their ears were placed on the disc-platform apparatus; and (e) the animals with the expandable foam or plastic tubes being removed from their ears were placed in the disc-platform apparatus. All of these behavioral experiments were performed in a quiet, enclosed darkroom, thus precluding any impact of vision on animals’ behavior.

Each of the four Asian shrew mole species *U. gracilis* (*N* = 16), *U. Soricipes* (*N* = 14), *U. nivatus* (*N = 11*), and *U. investigator* (*N* = 7) was individually tested for echolocation. Each of the behavioral experiments above required four trials for each individual to ensure the reliability and repeatability of the results. The emitted ultrasonic vocalizations were recorded using the microphone fixed in one of eight small discs and the platform, respectively. The position of the microphone on the small-disc ring apparatus was determined randomly, and the same position was set in the disc-platform apparatus for each trial. The infrared camera installed directly above the apparatus was used to perform video recordings during behavioral experiments. The audio and video recordings were analyzed synchronously to examine how the animals emitted ultrasonic calls during their movements in the apparatus. We analyzed video recordings with the VisuTrack software (Shanghai XinRuan Information Technology Co., Ltd.) that automatically documented the time when the animals entered and left the monitored sector, as well as the total exploration time on the center disc. The ultrasonic emissions in the monitored sector and its adjacent sectors were counted from the audio recordings using the software and methods above. We calculated the percentages of exploration time and pulse rate when the animals were exploring in the monitored sector. The data from the behavioral experiments above are shown in Table S3.

### Micro-computed tomography (μCT) imaging

Images of the thyroid cartilages were taken with a stereomicroscope (SMZ18, Nikon). μCT images of the spatial relationship between the stylohyal and tympanic bones were obtained for three Asian shrew mole species (*U. gracilis, U. investigator*, and *U. Soricipes*). The μCT scanner (Bruker Skyscan 1176) was used to collect images with a resolution of 9 μm at 65 kV and 310 μA. We used three-dimensional analysis software (CT-VOX) to reconstruct and visualize the data.

### Verifying the tongue as a vocal organ for echolocation in Asian shrew moles

To verify the tongue as a vocal organ for echolocation, we recorded ultrasonic vocalizations of Asian shrew moles while controlling the movements of the tongue. After the animals were completely anesthetized with isoflurane using an anesthesia machine (Reward RWD500), we tied a knot in the middle of the tongue with the surgical suture (0.2 mm). The ligation was removed after behavioral experiments. We used the same methods to treat the rousette bat (*R. leschenaultii*) and the soft-furred tree mouse (*T. daloushanensis*). Because the rousette bat emitted echolocation calls with the tongue (*11*) and the soft-furred tree mouse with the larynx (*4*), these two kinds of animals were used as the positive and negative controls, respectively. We recorded ultrasonic vocalizations of Asian shrew moles (*U. gracilis*) and soft-furred tree mice in cages (25 × 25 × 25 cm), and the rousette bats flying in an empty room (3 × 3 × 3 m). Before, during, and after the tongue being ligated, we recorded ultrasonic vocalizations of each animal for 10 minutes, respectively. The experiments were repeated three times for each individual of each species to ensure that the results were reliable and repeatable. Finally, we placed Asian shrew moles with ligated tongues in the disc-platform apparatus to determine whether Asian shrew moles echolocate with the tongue. The data from the behavioral experiments above are shown in Tables S4 and S5.

## Supplementary Material

nwaf591_Supplemental_Files

## Data Availability

All data are available in the main text or the supplementary materials.
