# Supplementary material for: Evidence for echolocation in Asian shrew moles
Source: Natl Sci Rev. 2025 Dec 26;13(7):nwaf591. doi: 10.1093/nsr/nwaf591 (PMC13114868; doi:10.1093/nsr/nwaf591)
Supplement: nwaf591_Supplemental_Files [file nwaf591_supplemental_files.zip › Supplementary_figures.docx]

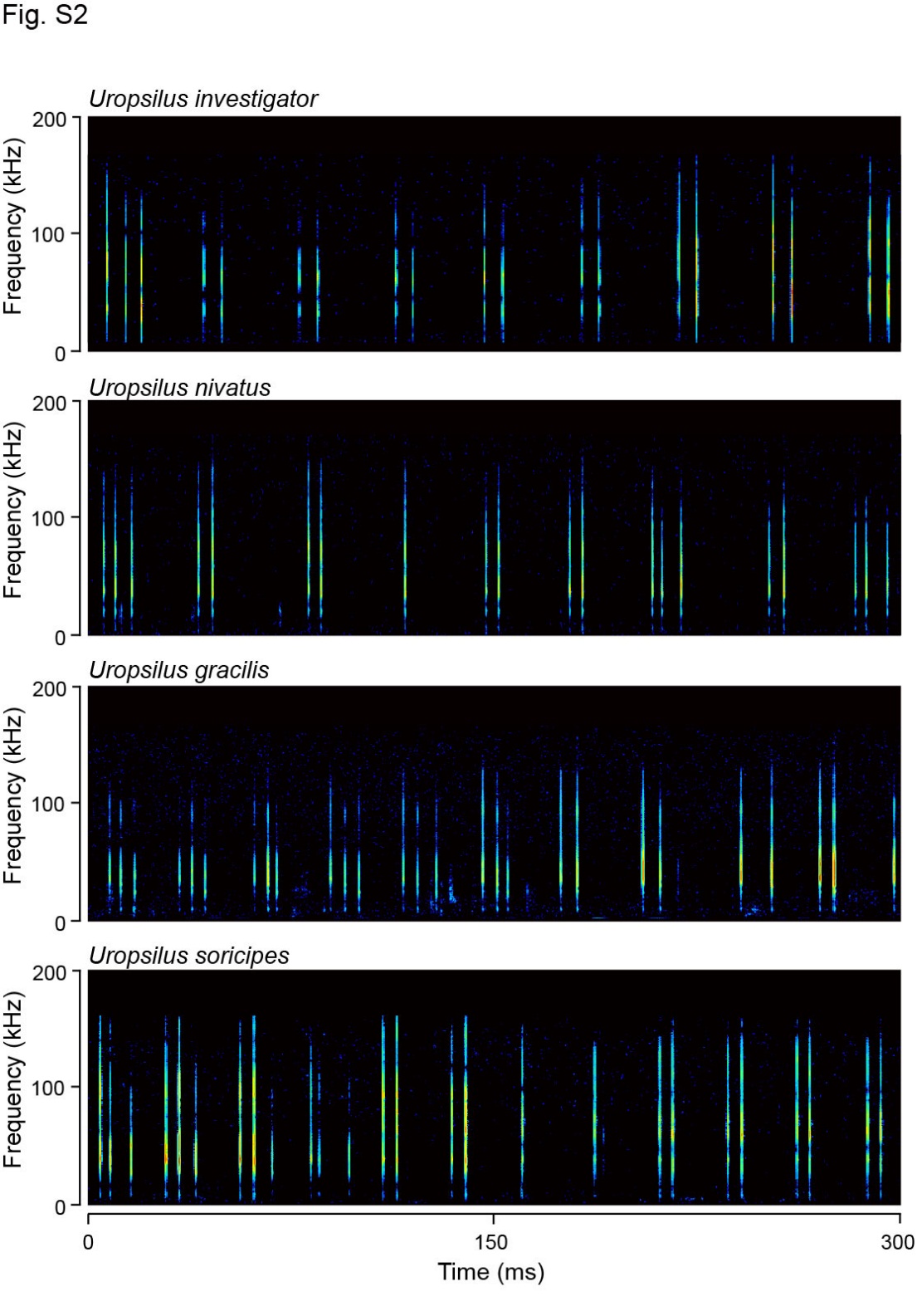


**Fig. S1.**

**Records of natural sequence of pulses emitted by four Asian shrew mole species.**

**
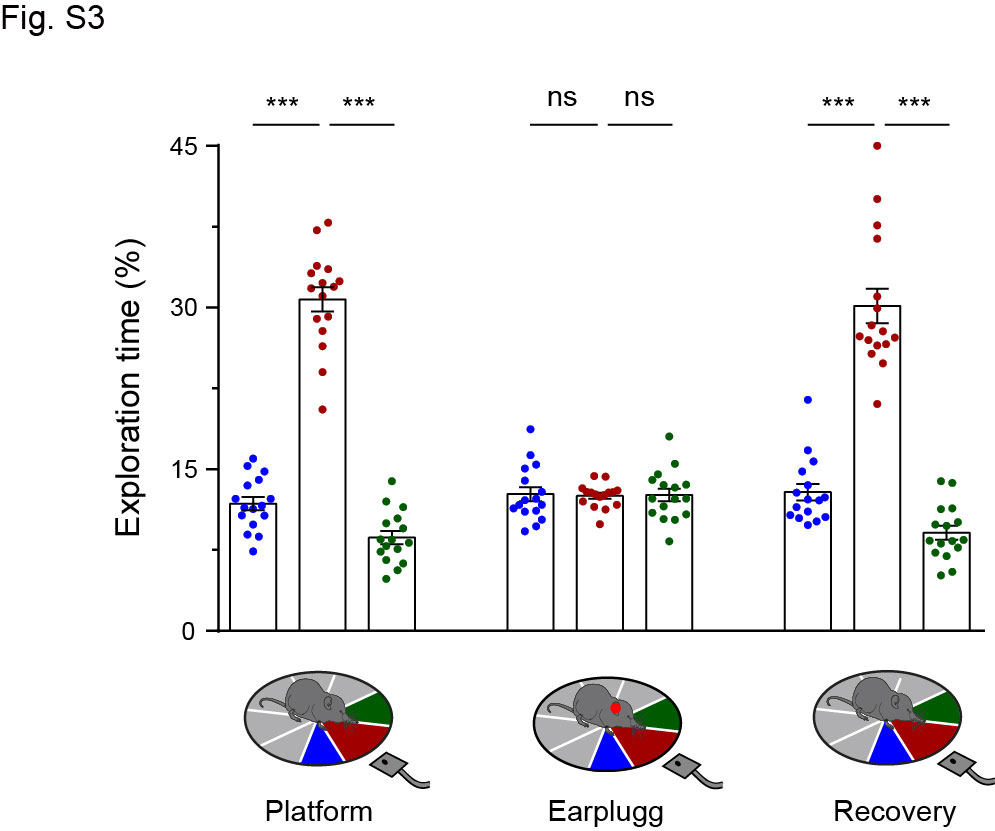
**

**Fig. S2.**

**Comparisons of the exploration time and pulse rate within the platform, earplug and recovery groups.** The percentage of exploration time was significantly larger when the gracile shrew moles (*U. gracilis*) explored in the monitored sector (red) than in its adjacent sectors (blue and green) in the platform and recovery groups. There was no significant difference in the percentage of exploration time when the gracile shrew moles explored in the monitored sector than in its adjacent sectors with earplugs. Each dot represents the mean of the measurements of each individual (n = 16 biological independent replicates). The bar denotes the mean value across eight individuals, and the error bars represent SEM. values. The *p* values are from two-tailed paired Student’s t tests. *** *p* < 0.001. ns means not significant.


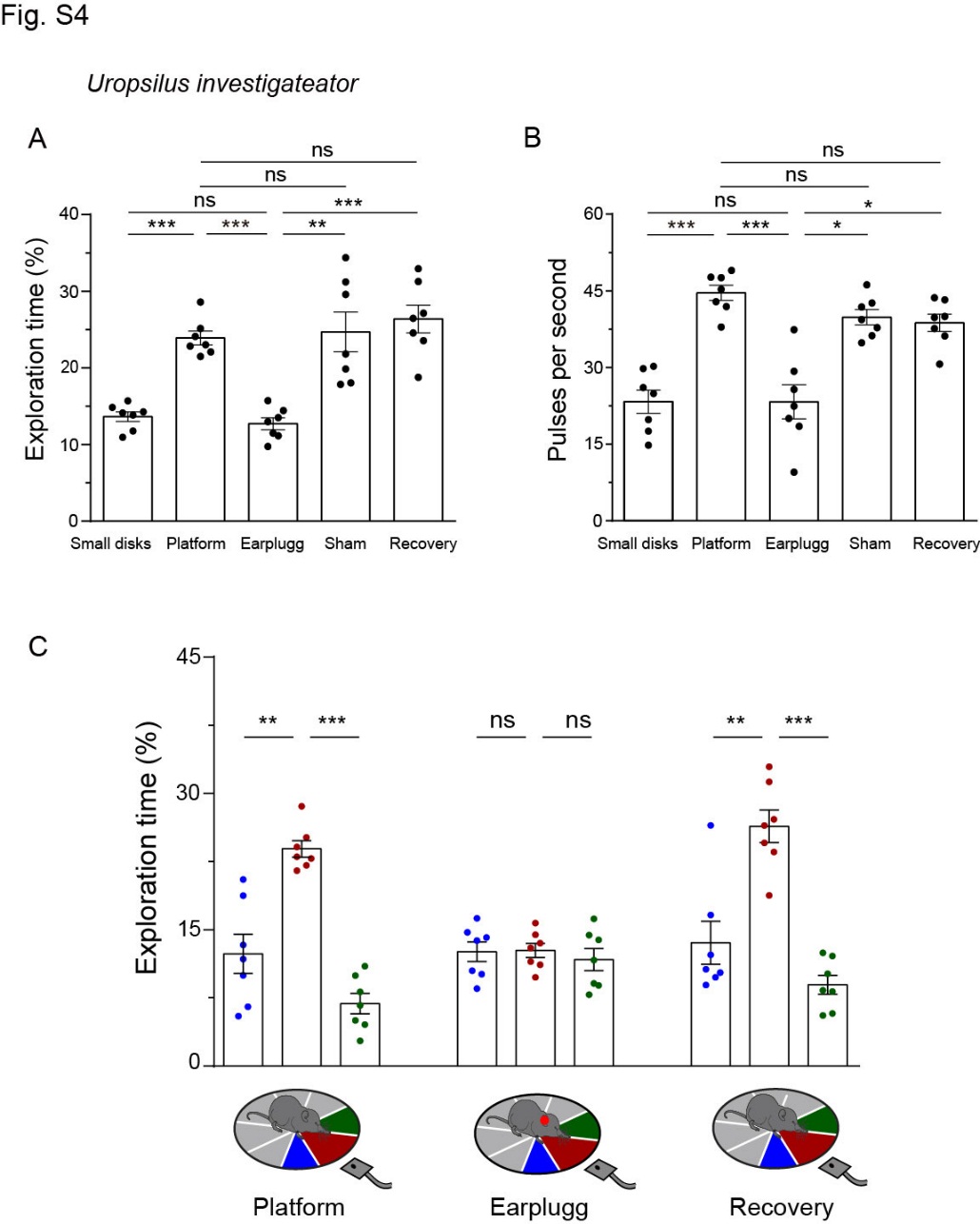


**Fig. S3.**

**Behavioral experiments for testing echolocation in *U.* investigateator.**

Comparisons of the percentage of exploration time (A) and pulse rates (B) in the sector corresponding to the ultrasonic probe under various experimental treatments when *U.* *investigator* explored the central disc. (C) The percentage of exploration time when *U. investigator* exploring in the monitored sector is also compared with its adjacent sectors within platform, earplug and recovery groups. The inset shows the monitored sector (red) and its adjacent (blue and green) sectors. Each dot represents the mean of the measurements of each individual (N = 7). The raw data points are shown in **Table S3**. The bar denotes the mean value across all individuals, and the error bars represent SEM. *P* values are from two-tailed paired Student’s t tests and were corrected with the Bonferroni method. * *p* < 0.05; ** *p* < 0.01; *** *p* < 0.001. ns, not significant.


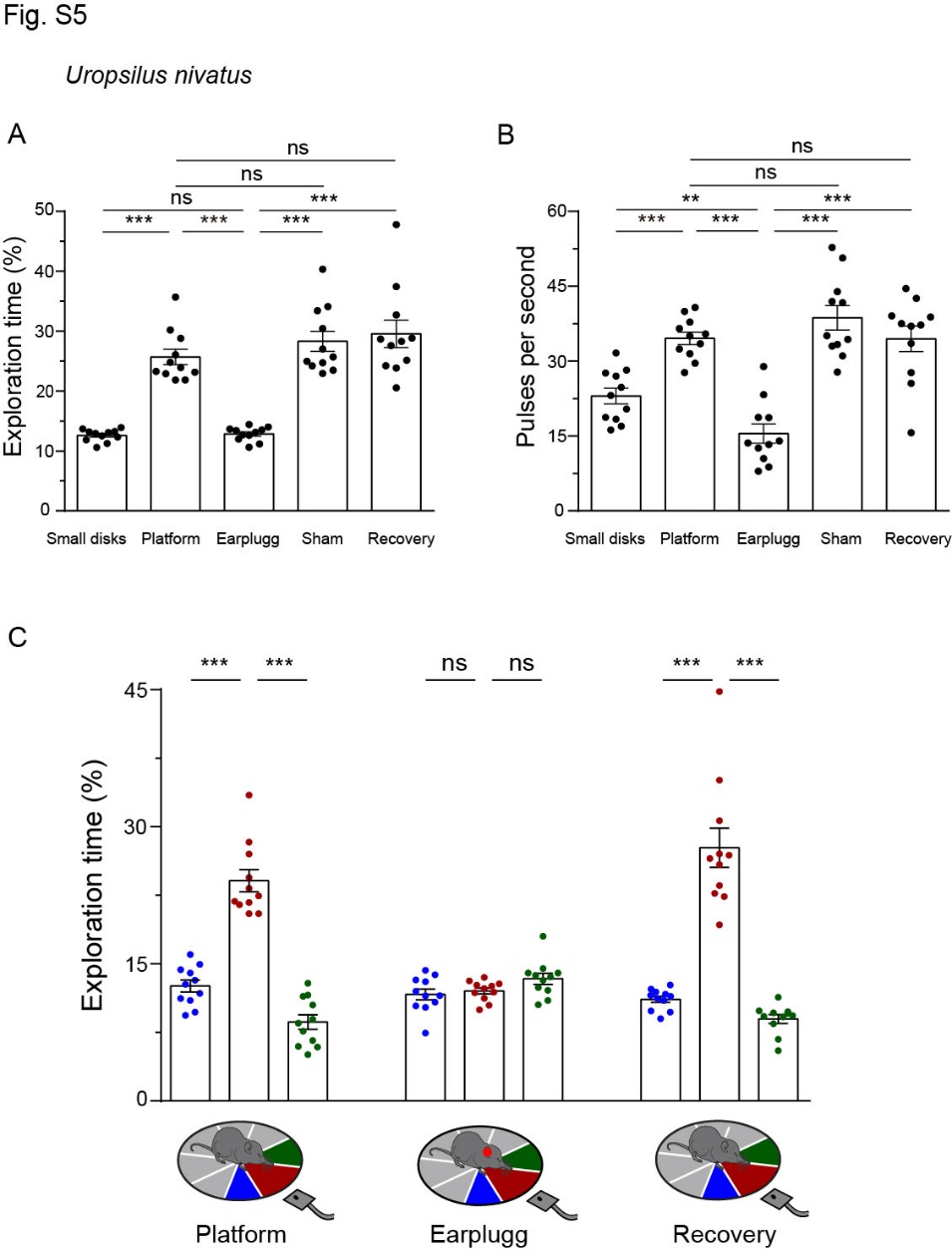


**Fig. S4.**

**Behavioral experiments for testing echolocation in *U. nivatus*.** Comparisons of the percentage of exploration time (A) and pulse rates (B) in the sector corresponding to the ultrasonic probe under various experimental treatments when *U. nivatus* explored the central disc. (C) The percentage of exploration time when *U. nivatus* explored the monitored sector is also compared with its adjacent sectors within the platform, earplug and recovery groups. The inset shows the monitored sector (red) and its adjacent (blue and green) sectors. Each dot represents the mean of the measurements of each individual (N = 11). The raw data points are shown in **Table S3**. The bar denotes the mean value across all individuals, and the error bars represent SEM. *P* values are from two-tailed paired Student’s t tests and were corrected with the Bonferroni method. * *p* < 0.05; ** *p* < 0.01; *** *p* < 0.001. ns, not significant.


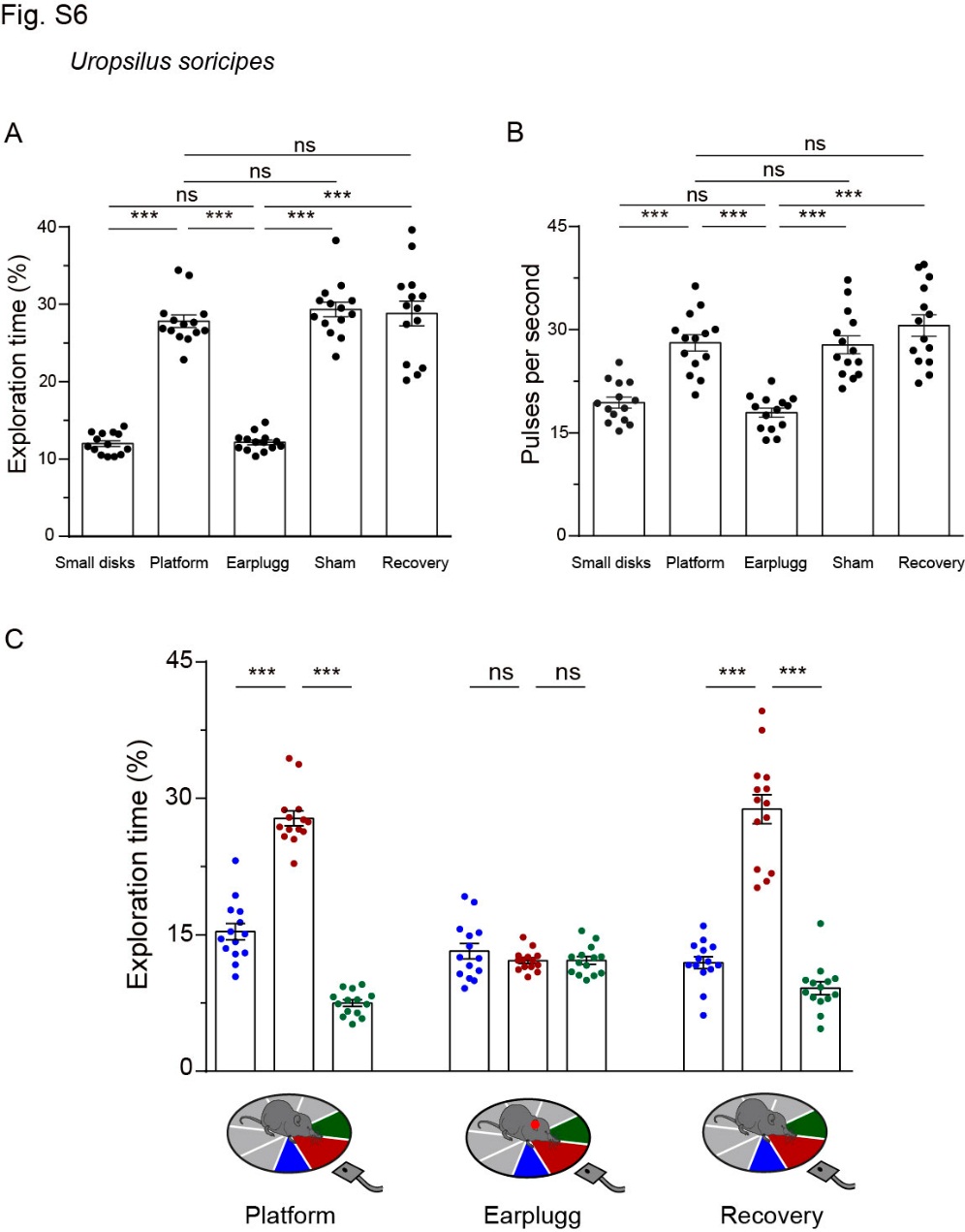


**Fig. S5.**

**Behavioral experiments for testing echolocation in *U.*** ***soricipes*.** Comparisons of the percentage of exploration time (A) and pulse rates (B) in the sector corresponding to the ultrasonic probe under various experimental treatments when *U.* *soricipes* explored the central disc. (C) The percentage of exploration time when *U.* *Soricipes* explored in the monitored sector is also compared with its adjacent sectors within the platform, earplug and recovery groups. The inset shows the monitored sector (red) and its adjacent (blue and green) sectors. Each dot represents the mean of the measurements of each individual (N = 14). The raw data points are shown in **Table S3**. The bar denotes the mean value across all individuals, and the error bars represent SEM. *P* values are from two-tailed paired Student’s t tests and were corrected with the Bonferroni method. * *p* < 0.05; ** *p* < 0.01; *** *p* < 0.001. ns, not significant.


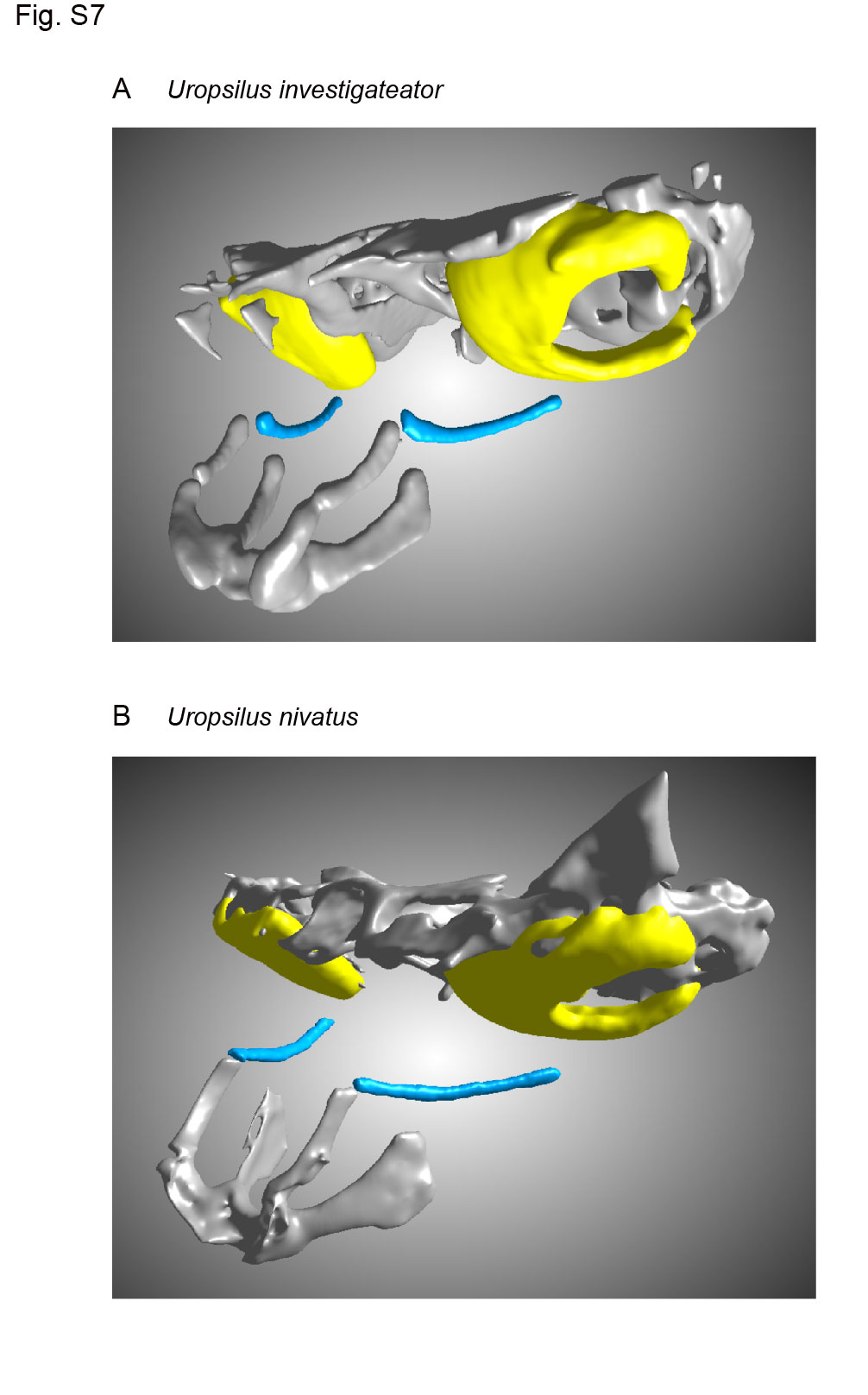


**Fig. S6.**

**Stylohyal and tympanic bones in *U.* investigateator (A) and *U. nivatus* (B).**
