# Supplementary material for: Evidence for echolocation in Asian shrew moles
Source: Natl Sci Rev. 2025 Dec 26;13(7):nwaf591. doi: 10.1093/nsr/nwaf591 (PMC13114868; doi:10.1093/nsr/nwaf591)
Supplement: nwaf591_Supplemental_Files [file nwaf591_supplemental_files.zip › Table_S1-6.pdf]

**Table S1. Acoustic variables of ultrasonic pulses from *Uropsilus* species.**

| Species / Acoustic parameters | *U. gracilis* | *U. investigator* | *U. nivatus* | *U. Soricipes* |
|---|---|---|---|---|
| N | 16 | 7 | 11 | 14 |
| n | 480 | 210 | 330 | 420 |
| Pulse duration (ms) | 0.80± 0.04 (0.4-1.23) | 1.05 ± 0.06 (0.97-1.41) | 0.77 ± 0.05 (0.46-1.53) | 0.63 ± 0.02 (0.55-1.75) |
| Pulse interval between two single pulse groups (ms) | 31.74 ± 0.64 (28.14-31.74) | 31.74 ± 2.15 (22.07-38.72) | 30.54 ± 0.33 (23.41-37.09 | 36.66 ± 2.16 (29.47-48.94) |
| Pulse interval within dyad pulse group (ms) | 3.14 ± 0.03 (1.78-7.2) | 3.18 ± 0.22 (2.08-6.68) | 3.18 ± 0.22 (2.08-6.68) | 3.68 ± 0.35 (2.21-6.20) |
| Pulse interval within triad pulse group (ms) | 2.85 ± 0.21 (2.32-4.44) | 2.67 ± 0.31 (1.52-3.57) | 2.67 ± 0.31 (1.52-3.57) | 3.17 ± 0.19 (2.43-4.87) |
| $f_{max}$ (kHz) | 107.68 ± 1.83 (79-147) | 92.86 ± 0.82 (90.49-95.29) | 100.47 ± 2.85 (71.40-125.90) | 102.43 ± 2.27 (91.43-113.95) |
| $f_{min}$ (kHz) | 42.2 ± 1.38 (22-68) | 30.96 ± 2.41 (22.26-40.93) | 26.19 ± 0.74 (20.50-33.30) | 28.24 ± 1.63 (22.42-42.71) |
| Bandwidth (kHz) | 65.48 ± 0.56 (56-79) | 61.86 ± 2.58 (51.65-70.15) | 74.23 ± 2.72 (43.50-90.20) | 74.15 ± 2.33 (67.84-86.72) |
| Peak frequency (kHz) | 63.03 ± 1.37 (42-89) | 57.66 ± 1.49 (51.94-64.56) | 47.63 ± 1.05 (35.71-56.20) | 57.85 ± 1.31 (51.27-65.72) |

N: Number of individuals
n: Number of pulses
Results are presented as Mean ± SEM (range).

**Table S2. Descriptive statistics for acoustic variables of ultrasonic pulses from echolocating and non-echolocation species.**

| Order | Suborder | Family | Scientific name | Duration (ms) | Minimum frequency (kHz) | Minimum frequency (kHz) | Bandwidth (kHz) | Peak frequency (kHz) | Echolocation calls type | Reference |
|---|---|---|---|---|---|---|---|---|---|---|
| Artiodactyla | Odontoceti | Delphinidae | *Cephalorhynchus commersonii* | 0.096 | 29 | 181 | 152 | 122 | click | Erbe etal. 2024 |
| Artiodactyla | Odontoceti | Delphinidae | *Cephalorhynchus eutropia* | 0.083 | 109 | 143 | 34 | 130 | click | Erbe etal. 2024 |
| Artiodactyla | Odontoceti | Delphinidae | *Cephalorhynchus heavisidii* | 0.06 | 12 | 141 | 129 | 126 | click | Erbe etal. 2024 |
| Artiodactyla | Odontoceti | Delphinidae | *Cephalorhynchus hectori* | 0.06 | 117 | 135 | 18 | 129 | click | Erbe etal. 2024 |
| Artiodactyla | Odontoceti | Delphinidae | *Delphinus delphis* | 0.125 | 20 | 190 | 170 | 75 | click | Erbe etal. 2024 |
| Artiodactyla | Odontoceti | Delphinidae | *Feresa attenuata* | 0.03 | 20 | 120 | 100 | 70 | click | Erbe etal. 2024 |
| Artiodactyla | Odontoceti | Delphinidae | *Globicephala macrorhynchus* | 0.301 | 7.8 | 55 | 47.2 | 30.5 | click | Erbe etal. 2024 |
| Artiodactyla | Odontoceti | Delphinidae | *Grampus griseus* | 0.058 | 2 | 110 | 108 | 44.9 | click | Erbe etal. 2024 |
| Artiodactyla | Odontoceti | Delphinidae | *Lagenorhynchus albirostris* | 0.023 | 41 | 122 | 81 | 82.2 | click | Erbe etal. 2024 |
| Artiodactyla | Odontoceti | Delphinidae | *Lagenorhynchus australis* | 0.092 | 120 | 133 | 13 | 126 | click | Erbe etal. 2024 |
| Artiodactyla | Odontoceti | Delphinidae | *Lagenorhynchus cruciger* | 0.115 | 122 | 131 | 9 | 126 | click | Erbe etal. 2024 |
| Artiodactyla | Odontoceti | Delphinidae | *Lagenorhynchus obliquidens* | 0.059 | 22 | 80 | 58 | 52.3 | click | Erbe etal. 2024 |
| Artiodactyla | Odontoceti | Delphinidae | *Lagenorhynchus obscurus* | 0.05 | 30 | 130 | 100 | 74 | click | Erbe etal. 2024 |
| Artiodactyla | Odontoceti | Delphinidae | *Lissodelphis borealis* | 0.31 | 23 | 41 | 18 | 31 | click | Erbe etal. 2024 |
| Artiodactyla | Odontoceti | Delphinidae | *Orcaella brevirostris* | 0.019 | 30 | 130 | 100 | 79.8 | click | Erbe etal. 2024 |
| Artiodactyla | Odontoceti | Delphinidae | *Orcaella heinsohni* | 0.012 | 6 | 123 | 117 | 37 | click | Erbe etal. 2024 |
| Artiodactyla | Odontoceti | Delphinidae | *Orcinus orca* | 0.098 | 10 | 108 | 98 | 28.8 | click | Erbe etal. 2024 |
| Artiodactyla | Odontoceti | Delphinidae | *Peponocephala electra* | 0.324 | 13 | 41 | 28 | 26.2 | click | Erbe etal. 2024 |
| Artiodactyla | Odontoceti | Delphinidae | *Pseudorca crassidens* | 0.089 | 7.8 | 111 | 103.2 | 50.2 | click | Erbe etal. 2024 |
| Artiodactyla | Odontoceti | Delphinidae | *Sotalia fluviatilis* | 0.04 | 47 | 137 | 90 | 90.3 | click | Erbe etal. 2024 |
| Artiodactyla | Odontoceti | Delphinidae | *Sotalia guianensis* | 0.05 | 9.3 | 41 | 31.7 | 25.2 | click | Erbe etal. 2024 |
| Artiodactyla | Odontoceti | Delphinidae | *Sousa chinensis* | 0.021 | 30 | 200 | 170 | 109 | click | Erbe etal. 2024 |
| Artiodactyla | Odontoceti | Delphinidae | *Stenella attenuata* | 0.031 | 38 | 111 | 73 | 79.5 | click | Erbe etal. 2024 |
| Artiodactyla | Odontoceti | Delphinidae | *Stenella frontalis* | 0.037 | 30 | 109 | 79 | 59 | click | Erbe etal. 2024 |
| Artiodactyla | Odontoceti | Delphinidae | *Stenella longirostris* | 0.191 | 20 | 130 | 110 | 59.2 | click | Erbe etal. 2024 |
| Artiodactyla | Odontoceti | Delphinidae | *Steno bredanensis* | 0.23 | 15 | 112 | 97 | 43.5 | click | Erbe etal. 2024 |
| Artiodactyla | Odontoceti | Delphinidae | *Tursiops aduncus* | 0.015 | 45 | 141 | 96 | 96 | click | Erbe etal. 2024 |
| Artiodactyla | Odontoceti | Delphinidae | *Tursiops truncatus* | 0.091 | 7 | 205 | 198 | 76.4 | click | Erbe etal. 2024 |
| Artiodactyla | Odontoceti | Lipotidae | *Lipotes vexillifer* | 0.018 | 50 | 120 | 70 | 75 | click | Erbe etal. 2024 |
| Artiodactyla | Odontoceti | Monodontidae | *Delphinapterus leucas* | 0.075 | 40 | 120 | 80 | 80 | click | Erbe etal. 2024 |
| Artiodactyla | Odontoceti | Monodontidae | *Monodon monoceros* | 0.029 | 0.5 | 112 | 111.5 | 32.1 | click | Erbe etal. 2024 |
| Artiodactyla | Odontoceti | Iniidae | *Inia geoffrensis* | 0.037 | 23.1 | 170 | 146.9 | 71.8 | click | Erbe etal. 2024 |
| Artiodactyla | Odontoceti | Kogiidae | *Kogia breviceps* | 0.28 | 60 | 200 | 140 | 128 | click | Erbe etal. 2024 |
| Artiodactyla | Odontoceti | Kogiidae | *Kogia sima* | 0.136 | 91 | 140 | 49 | 123 | click | Erbe etal. 2024 |
| Artiodactyla | Odontoceti | Phocoenidae | *Neophocaena asiaeorientalis* | 0.064 | 87 | 149 | 62 | 130 | click | Erbe etal. 2024 |

| | | | | | | | | | | |
|---|---|---|---|---|---|---|---|---|---|---|
| Artiodactyla | Odontoceti | Phocoenidae | *Phocoena phocoena* | 0.096 | 110 | 215 | 105 | 145 | click | Erbe etal. 2024 |
| Artiodactyla | Odontoceti | Phocoenidae | *Phocoena sinus* | 0.136 | 128 | 139 | 11 | 133 | click | Erbe etal. 2024 |
| Artiodactyla | Odontoceti | Phocoenidae | *Phocoena spinipinnis* | 0.144 | 129 | 186 | 57 | 138 | click | Erbe etal. 2024 |
| Artiodactyla | Odontoceti | Phocoenidae | *Phocoenoides dalli* | 0.204 | 90 | 149 | 59 | 129 | click | Erbe etal. 2024 |
| Artiodactyla | Odontoceti | Pontoporiidae | *Pontoporia blainvillei* | 2.737 | 11 | 149 | 138 | 80.5 | click | Erbe etal. 2024 |
| Artiodactyla | Odontoceti | Ziphiidae | *Tasmacetus shepherdi* | 0.27 | 12.2 | 25.9 | 13.7 | 19.3 | click | Erbe etal. 2024 |
| Artiodactyla | Odontoceti | Ziphiidae | *Mesoplodon bidens* | 0.318 | 27.4 | 76.5 | 49.1 | 49.6 | click | Erbe etal. 2024 |
| Artiodactyla | Odontoceti | Ziphiidae | *Mesoplodon densirostris* | 0.268 | 26 | 63 | 37 | 39.7 | click | Erbe etal. 2024 |
| Artiodactyla | Odontoceti | Ziphiidae | *Mesoplodon europaeus* | 0.325 | 30 | 55.9 | 25.9 | 41.9 | click | Erbe etal. 2024 |
| Artiodactyla | Odontoceti | Ziphiidae | *Mesoplodon mirus* | 0.255 | 35 | 55 | 20 | 41.7 | click | Erbe etal. 2024 |
| Artiodactyla | Odontoceti | Ziphiidae | *Mesoplodon stejnegeri* | 0.42 | 45.7 | 73.8 | 28.1 | 50.4 | click | Erbe etal. 2024 |
| Artiodactyla | Odontoceti | Ziphiidae | *Hyperoodon ampullatus* | 0.321 | 4.4 | 90 | 85.6 | 31.7 | click | Erbe etal. 2024 |
| Artiodactyla | Odontoceti | Ziphiidae | *Indopacetus pacificus* | 0.23 | 12.7 | 37.7 | 25 | 22.4 | click | Erbe etal. 2024 |
| Artiodactyla | Odontoceti | Ziphiidae | *Berardius bairdii* | 0.464 | 9 | 43 | 34 | 21.5 | click | Erbe etal. 2024 |
| Artiodactyla | Odontoceti | Ziphiidae | *Ziphius cavirostris* | 0.51 | 13 | 49.2 | 36.2 | 32.6 | click | Erbe etal. 2024 |
| Artiodactyla | Mysticeti | Balaenidae | *Balaena mysticetus* | 4100 | 0.288 | 2.031 | 1.743 | 0.679 | non-echolocation | Erbe etal. 2024 |
| Artiodactyla | Mysticeti | Balaenopteridae | *Balaenoptera acutorostrata* | 280 | 1 | 1.8 | 0.8 | 1.4 | non-echolocation | Erbe etal. 2024 |
| Artiodactyla | Mysticeti | Balaenopteridae | *Balaenoptera bonaerensis* | 400 | 0.133 | 0.229 | 0.096 | 0.174 | non-echolocation | Erbe etal. 2024 |
| Artiodactyla | Mysticeti | Balaenopteridae | *Balaenoptera edeni* | 1200 | 0.311 | 0.701 | 0.39 | 0.402 | non-echolocation | Erbe etal. 2024 |
| Artiodactyla | Mysticeti | Balaenopteridae | *Balaenoptera musculus* | 10000 | 0.049 | 0.102 | 0.053 | 0.06 | non-echolocation | Erbe etal. 2024 |
| Artiodactyla | Mysticeti | Balaenopteridae | *Balaenoptera omurai* | 13000 | 0.016 | 0.051 | 0.035 | 0.034 | non-echolocation | Erbe etal. 2024 |
| Artiodactyla | Mysticeti | Balaenopteridae | *Balaenoptera physalus* | 270 | 0.042 | 0.064 | 0.022 | 0.047 | non-echolocation | Erbe etal. 2024 |
| Artiodactyla | Mysticeti | Neobalaenidae | *Caperea marginata* | 180 | 0.06 | 0.135 | 0.075 | 0.07 | non-echolocation | Erbe etal. 2024 |
| Artiodactyla | Mysticeti | Balaenopteridae | *Eschrichtius robustus* | 830 | 0.141 | 1.21 | 1.069 | 0.342 | non-echolocation | Erbe etal. 2024 |
| Artiodactyla | Mysticeti | Balaenidae | *Eubalaena australis* | 1200 | 0.061 | 0.916 | 0.855 | 0.205 | non-echolocation | Erbe etal. 2024 |
| Artiodactyla | Mysticeti | Balaenidae | *Eubalaena glacialis* | 710 | 0.326 | 1.626 | 1.3 | 0.576 | non-echolocation | Erbe etal. 2024 |
| Artiodactyla | Mysticeti | Balaenidae | *Eubalaena japonica* | 1300 | 0.065 | 0.2 | 0.135 | 0.133 | non-echolocation | Erbe etal. 2024 |
| Chiroptera | Yangochiroptera | Emballonuridae | *Emballonura monticola* | 5.42 | 38.91 | 53.55 | 14.64 | 51.24 | FM | Luo et al. 2019 |
| Chiroptera | Yangochiroptera | Emballonuridae | *Peropteryx macrotis* | 7.3 | 37.54 | 41.87 | 4.33 | 41.61 | FM | Zamora-Gutierrez et al. 2016 |
| Chiroptera | Yangochiroptera | Emballonuridae | *Rhynchonycteris naso* | 4.38 | 81.84 | 98.71 | 16.86 | 95.79 | FM | Zamora-Gutierrez et al. 2016 |
| Chiroptera | Yangochiroptera | Emballonuridae | *Saccopteryx bilineata* | 9.7 | 44 | 46.4 | 2.4 | 45.7 | FM | Biscardi et al. 2004 |
| Chiroptera | Yangochiroptera | Emballonuridae | *Saccopteryx leptura* | 7.5 | 51.4 | 54.5 | 3.1 | 53.6 | FM | Biscardi et al. 2004 |
| Chiroptera | Yangochiroptera | Emballonuridae | *Taphozous melanopogon* | 6.02 | 22.58 | 36.6 | 14.02 | 29.71 | FM | Luo et al. 2019 |
| Chiroptera | Yangochiroptera | Miniopteridae | *Miniopterus australis* | 4.42 | 47.2 | 95.4 | 48.2 | 61.46 | FM | Luo et al. 2019 |
| Chiroptera | Yangochiroptera | Miniopteridae | *Miniopterus magnater* | 4.9 | 39 | 117.6 | 78.6 | 46.5 | FM | our data |
| Chiroptera | Yangochiroptera | Miniopteridae | *Miniopterus pusillus* | 4.65 | 52.28 | 106.17 | 53.89 | 62.85 | FM | Luo et al. 2019 |
| Chiroptera | Yangochiroptera | Molossidae | *Eumops underwoodi* | 14.22 | 15.4 | 18.09 | 2.69 | 16.57 | FM | Miller 2003 |

| | | | | | | | | | | |
|---|---|---|---|---|---|---|---|---|---|---|
| Chiroptera | Yangochiroptera | Molossidae | *Molossus coibensis* | 7.06 | 26.74 | 37.3 | 8.43 | 31.66 | CF | Collen 2012 |
| Chiroptera | Yangochiroptera | Molossidae | *Molossus molossus* | 8.72 | 34.71 | 38.93 | 4.22 | 38.38 | FM | Zamora-Gutierrez et al. 2016 |
| Chiroptera | Yangochiroptera | Molossidae | *Molossus rufus* | 9.73 | 28.79 | 32.28 | 3.49 | 31.89 | FM | Zamora-Gutierrez et al. 2016 |
| Chiroptera | Yangochiroptera | Molossidae | *Mops mops* | 16.7 | 15.5 | 25.9 | 10.4 | 18.5 | FM | Luo et al. 2019 |
| Chiroptera | Yangochiroptera | Molossidae | *Nyctinomops femorosaccus* | 6.16 | 38.5 | 67.4 | 28.9 | 42.44 | FM | Zamora-Gutierrez et al. 2016 |
| Chiroptera | Yangochiroptera | Molossidae | *Nyctinomops laticaudatus* | 4.85 | 17.96 | 41.03 | 23.07 | 25.68 | FM | Zamora-Gutierrez et al. 2016 |
| Chiroptera | Yangochiroptera | Molossidae | *Nyctinomops macrotis* | 7.92 | 13.79 | 28.66 | 14.86 | 22.34 | FM | Zamora-Gutierrez et al. 2016 |
| Chiroptera | Yangochiroptera | Molossidae | *Tadarida aegyptiaca* | 15.5 | 16.67 | 24.44 | 7.77 | 19.44 | FM | Luo et al. 2019 |
| Chiroptera | Yangochiroptera | Molossidae | *Tadarida brasiliensis* | 7.2 | 26.09 | 46.83 | 20.74 | 32.61 | FM | Zamora-Gutierrez et al. 2016 |
| Chiroptera | Yangochiroptera | Mormoopidae | *Mormoops megalophylla* | 4.78 | 42.1 | 55.66 | 13.56 | 52 | FM | Zamora-Gutierrez et al. 2016 |
| Chiroptera | Yangochiroptera | Mormoopidae | *Pteronotus davyi* | 6.7 | 69.2 | 79.2 | 10 | 71.5 | FM | Biscardi et al. 2004 |
| Chiroptera | Yangochiroptera | Mormoopidae | *Pteronotus gymnonotus* | 5.33 | 45.81 | 55.51 | 9.7 | 51.34 | FM | Zamora-Gutierrez et al. 2016 |
| Chiroptera | Yangochiroptera | Mormoopidae | *Pteronotus personatus* | 5.71 | 64.12 | 82.88 | 18.76 | 70.53 | FM | Zamora-Gutierrez et al. 2016 |
| Chiroptera | Yangochiroptera | Natalidae | *Natalus stramineus* | 2.11 | 94.99 | 156.14 | 61.15 | 119.4 | FM | Zamora-Gutierrez et al. 2016 |
| Chiroptera | Yangochiroptera | Noctilionidae | *Noctilio albiventris* | 10.5 | 52.35 | 85.08 | 27.4 | 69.5 | CF | Collen 2012 |
| Chiroptera | Yangochiroptera | Noctilionidae | *Noctilio leporinus* | 8.41 | 23.52 | 50.96 | 27.43 | 31.03 | CF | Zamora-Gutierrez et al. 2016 |
| Chiroptera | Yangochiroptera | Nycteridae | *Nycteris tragata* | 2.87 | 71.13 | 111.88 | 40.75 | 97.64 | FM | Luo et al. 2019 |
| Chiroptera | Yangochiroptera | Nycteridae | *Nycticeius humeralis* | 5.55 | 17.08 | 36.55 | 19.46 | 24.38 | FM | Zamora-Gutierrez et al. 2016 |
| Chiroptera | Yangochiroptera | Phyllostomidae | *Anoura geoffroyi* | 2.08 | 66.3 | 105.87 | 39.57 | 83.08 | FM | Zamora-Gutierrez et al. 2016 |
| Chiroptera | Yangochiroptera | Phyllostomidae | *Artibeus jamaicensis* | 2.45 | 44.01 | 69.25 | 25.24 | 57.04 | FM | Zamora-Gutierrez et al. 2016 |
| Chiroptera | Yangochiroptera | Phyllostomidae | *Artibeus lituratus* | 2.04 | 49.92 | 76.68 | 26.76 | 61.44 | FM | Zamora-Gutierrez et al. 2016 |
| Chiroptera | Yangochiroptera | Phyllostomidae | *Brachyphylla cavernarum* | 2.6 | 38 | 66.8 | 28.8 | 51.4 | FM | Luo et al. 2019 |
| Chiroptera | Yangochiroptera | Phyllostomidae | *Carollia perspicillata* | 2.42 | 45.67 | 75.37 | 29.69 | 62.66 | FM | Zamora-Gutierrez et al. 2016 |
| Chiroptera | Yangochiroptera | Phyllostomidae | *Desmodus rotundus* | 2.54 | 43.98 | 72.35 | 28.37 | 56.87 | FM | Zamora-Gutierrez et al. 2016 |
| Chiroptera | Yangochiroptera | Phyllostomidae | *Erophylla bombifrons* | 4.7 | 26.8 | 54.2 | 27.4 | 37.9 | FM | Luo et al. 2019 |
| Chiroptera | Yangochiroptera | Phyllostomidae | *Leptonycteris yerbabuenae* | 6.22 | 34.12 | 79.28 | 45.16 | 49.72 | FM | Zamora-Gutierrez et al. 2016 |
| Chiroptera | Yangochiroptera | Phyllostomidae | *Macrotus californicus* | 3.31 | 47.45 | 80.44 | 32.99 | 60.39 | FM | Zamora-Gutierrez et al. 2016 |
| Chiroptera | Yangochiroptera | Phyllostomidae | *Phyllops falcatus* | 4.2 | 23.9 | 73.9 | 50 | 56.2 | FM | Luo et al. 2019 |
| Chiroptera | Yangochiroptera | Phyllostomidae | *Stenoderma rufum* | 3.1 | 26 | 95 | 69 | 67.6 | FM | Luo et al. 2019 |
| Chiroptera | Yangochiroptera | Phyllostomidae | *Sturnira lilium* | 3.13 | 52.62 | 90.86 | 38.24 | 72.44 | FM | Zamora-Gutierrez et al. 2016 |
| Chiroptera | Yangochiroptera | Phyllostomidae | *Sturnira ludovici* | 3.99 | 49.06 | 82.43 | 33.37 | 68.65 | FM | Zamora-Gutierrez et al. 2016 |
| Chiroptera | Yangochiroptera | Thyropteridae | *Thyroptera tricolor* | 2.76 | 43.5 | 66.38 | 22.88 | 53.09 | FM | Zamora-Gutierrez et al. 2016 |
| Chiroptera | Yangochiroptera | Vespertilionidae | *Antrozous pallidus* | 5.63 | 28.21 | 62.23 | 34.02 | 36.46 | FM | Zamora-Gutierrez et al. 2016 |
| Chiroptera | Yangochiroptera | Vespertilionidae | *Arielulus circumdatus* | 3.7 | 37.7 | 100.6 | 62.9 | 62.3 | FM | our data |
| Chiroptera | Yangochiroptera | Vespertilionidae | *Barbastella barbastellus* | 3.4 | 28 | 39.4 | 11.4 | 33.2 | FM | Luo et al. 2019 |

| | | | | | | | | | | |
|---|---|---|---|---|---|---|---|---|---|---|
| Chiroptera | Yangochiroptera | Vespertilionidae | *Corynorhinus mexicanus* | 3.66 | 23.77 | 45.07 | 21.31 | 34.92 | FM | Zamora-Gutierrez et al. 2016 |
| Chiroptera | Yangochiroptera | Vespertilionidae | *Corynorhinus townsendii* | 2.27 | 24.47 | 41.53 | 17.06 | 33.75 | FM | Zamora-Gutierrez et al. 2016 |
| Chiroptera | Yangochiroptera | Vespertilionidae | *Eptesicus bottae* | 6.19 | 30.51 | 42.63 | 12.12 | 32.08 | FM | Luo et al. 2019 |
| Chiroptera | Yangochiroptera | Vespertilionidae | *Eptesicus brasiliensis* | 7.8 | 32.93 | 55.83 | 22.89 | 37.12 | FM | Zamora-Gutierrez et al. 2016 |
| Chiroptera | Yangochiroptera | Vespertilionidae | *Eptesicus nilssonii* | 6.3 | 26.1 | 57.9 | 31.8 | 30.5 | FM | Luo et al. 2019 |
| Chiroptera | Yangochiroptera | Vespertilionidae | *Eptesicus serotinus* | 7.3 | 27.1 | 50.4 | 23.3 | 29.9 | FM | Luo et al. 2019 |
| Chiroptera | Yangochiroptera | Vespertilionidae | *Ia io* | 3.8 | 18 | 38.6 | 20.6 | 24.8 | FM | Luo et al. 2019 |
| Chiroptera | Yangochiroptera | Vespertilionidae | *Idionycteris phyllotis* | 4.28 | 13.83 | 30 | 16.17 | 21.04 | FM | Zamora-Gutierrez et al. 2016 |
| Chiroptera | Yangochiroptera | Vespertilionidae | *Kerivoula hardwickii* | 3.15 | 90.73 | 169.57 | 78.84 | 118.25 | FM | Luo et al. 2019 |
| Chiroptera | Yangochiroptera | Vespertilionidae | *Kerivoula kachinensis* | 3.3 | 84 | 132 | 48 | 109.2 | FM | our data |
| Chiroptera | Yangochiroptera | Vespertilionidae | *Lasionycteris noctivagans* | 3.29 | 39.49 | 90.03 | 50.54 | 53.75 | FM | Zamora-Gutierrez et al. 2016 |
| Chiroptera | Yangochiroptera | Vespertilionidae | *Lasiurus blossevillii* | 8.83 | 37.84 | 55.45 | 17.62 | 40.36 | FM | Zamora-Gutierrez et al. 2016 |
| Chiroptera | Yangochiroptera | Vespertilionidae | *Lasiurus borealis* | 4.25 | 26.79 | 57.24 | 30.45 | 35.47 | FM | Zamora-Gutierrez et al. 2016 |
| Chiroptera | Yangochiroptera | Vespertilionidae | *Lasiurus varius* | 7.64 | 33.18 | 52.25 | 19.07 | 36.44 | FM | Luo et al. 2019 |
| Chiroptera | Yangochiroptera | Vespertilionidae | *Murina suilla* | 2.91 | 82.42 | 142.62 | 60.2 | 101.93 | FM | Luo et al. 2019 |
| Chiroptera | Yangochiroptera | Vespertilionidae | *Myotis auriculus* | 4.62 | 31.5 | 76.33 | 44.83 | 43.74 | FM | Zamora-Gutierrez et al. 2016 |
| Chiroptera | Yangochiroptera | Vespertilionidae | *Myotis bechsteinii* | 4.6 | 35.6 | 92.8 | 57.2 | 44.3 | FM | Luo et al. 2019 |
| Chiroptera | Yangochiroptera | Vespertilionidae | *Myotis brandtii* | 3.06 | 33.7 | 85.5 | 51.8 | 47.9 | FM | Luo et al. 2019 |
| Chiroptera | Yangochiroptera | Vespertilionidae | *Myotis californicus* | 3.74 | 41.87 | 88.85 | 46.98 | 55.18 | FM | Zamora-Gutierrez et al. 2016 |
| Chiroptera | Yangochiroptera | Vespertilionidae | *Myotis capaccinii* | 4.6 | 35.1 | 89.7 | 54.6 | 48.9 | FM | Luo et al. 2019 |
| Chiroptera | Yangochiroptera | Vespertilionidae | *Myotis chinensis* | 3.6 | 25.2 | 83.6 | 58.4 | 49 | FM | Luo et al. 2019 |
| Chiroptera | Yangochiroptera | Vespertilionidae | *Myotis daubentonii* | 4.2 | 30.5 | 88 | 57.5 | 49.3 | FM | Luo et al. 2019 |
| Chiroptera | Yangochiroptera | Vespertilionidae | *Myotis emarginatus* | 3.6 | 41.2 | 109 | 67.8 | 58 | FM | Luo et al. 2019 |
| Chiroptera | Yangochiroptera | Vespertilionidae | *Myotis horsfieldii* | 3.17 | 39.63 | 87.25 | 47.62 | 56.93 | FM | Luo et al. 2019 |
| Chiroptera | Yangochiroptera | Vespertilionidae | *Myotis keaysi* | 3.3 | 58.71 | 88.47 | 29.76 | 60.87 | FM | Zamora-Gutierrez et al. 2016 |
| Chiroptera | Yangochiroptera | Vespertilionidae | *Myotis longipes* | 3.5 | 62.9 | 90.7 | 27.8 | 68.2 | FM | our data |
| Chiroptera | Yangochiroptera | Vespertilionidae | *Myotis macrodactylus* | 6.27 | 42.31 | 73.25 | 30.94 | 49.57 | FM | Luo et al. 2019 |
| Chiroptera | Yangochiroptera | Vespertilionidae | *Myotis myotis* | 4.6 | 27.9 | 79.6 | 51.7 | 39.1 | FM | Luo et al. 2019 |
| Chiroptera | Yangochiroptera | Vespertilionidae | *Myotis mystacinus* | 4.2 | 32.4 | 96.4 | 64 | 47.5 | FM | Luo et al. 2019 |
| Chiroptera | Yangochiroptera | Vespertilionidae | *Myotis nattereri* | 4.7 | 24.4 | 111.8 | 87.4 | 46.9 | FM | Luo et al. 2019 |
| Chiroptera | Yangochiroptera | Vespertilionidae | *Myotis nigricans* | 5.75 | 51.25 | 78.45 | 27.2 | 54.6 | FM | Luo et al. 2019 |
| Chiroptera | Yangochiroptera | Vespertilionidae | *Myotis thysanodes* | 3.71 | 17.29 | 68.92 | 51.63 | 29.02 | FM | Zamora-Gutierrez et al. 2016 |
| Chiroptera | Yangochiroptera | Vespertilionidae | *Myotis velifer* | 4.67 | 38.12 | 80.7 | 42.58 | 46.23 | FM | Zamora-Gutierrez et al. 2016 |
| Chiroptera | Yangochiroptera | Vespertilionidae | *Myotis volans* | 5.12 | 37.11 | 84.39 | 47.29 | 47.64 | FM | Zamora-Gutierrez et al. 2016 |
| Chiroptera | Yangochiroptera | Vespertilionidae | *Myotis yumanensis* | 3.69 | 42.76 | 87.56 | 44.8 | 52.64 | FM | Zamora-Gutierrez et al. 2016 |

| | | | | | | | | | | |
|---|---|---|---|---|---|---|---|---|---|---|
| Chiroptera | Yangochiroptera | Vespertilionidae | *Nyctalus leisleri* | 5.3 | 27.7 | 55 | 27.3 | 30.7 | FM | Luo et al. 2019 |
| Chiroptera | Yangochiroptera | Vespertilionidae | *Otonycteris hemprichii* | 5.19 | 20.94 | 36.86 | 15.92 | 24.45 | FM | Luo et al. 2019 |
| Chiroptera | Yangochiroptera | Vespertilionidae | *Pipistrellus abramus* | 7.94 | 43.11 | 55.4 | 12.29 | 44.81 | FM | Luo et al. 2019 |
| Chiroptera | Yangochiroptera | Vespertilionidae | *Pipistrellus kuhlii* | 6.4 | 37.5 | 59.8 | 22.3 | 39.7 | FM | Luo et al. 2019 |
| Chiroptera | Yangochiroptera | Vespertilionidae | *Pipistrellus maderensis* | 6.7 | 43.7 | 50.9 | 7.2 | 44.6 | FM | Luo et al. 2019 |
| Chiroptera | Yangochiroptera | Vespertilionidae | *Pipistrellus nathusii* | 6.2 | 38.7 | 75.7 | 37 | 40.8 | FM | Luo et al. 2019 |
| Chiroptera | Yangochiroptera | Vespertilionidae | *Pipistrellus pipistrellus* | 5.9 | 46.6 | 68.8 | 22.2 | 46.9 | FM | Luo et al. 2019 |
| Chiroptera | Yangochiroptera | Vespertilionidae | *Pipistrellus pygmaeus* | 5.5 | 56.8 | 79.6 | 22.8 | 57.7 | FM | Luo et al. 2019 |
| Chiroptera | Yangochiroptera | Vespertilionidae | *Pipistrellus stenopterus* | 13.8 | 28 | 42.8 | 14.8 | 31 | FM | Luo et al. 2019 |
| Chiroptera | Yangochiroptera | Vespertilionidae | *Plecotus auritus* | 2.3 | 26 | 44.7 | 18.7 | 33.1 | FM | Luo et al. 2019 |
| Chiroptera | Yangochiroptera | Vespertilionidae | *Plecotus austriacus* | 3.8 | 23.6 | 41.4 | 17.8 | 32.6 | FM | Luo et al. 2019 |
| Chiroptera | Yangochiroptera | Vespertilionidae | *Plecotus macrobullaris* | 3.98 | 43.95 | 19.35 | 24.6 | 28.53 | FM | Luo et al. 2019 |
| Chiroptera | Yangochiroptera | Vespertilionidae | *Rhogeessa aeneus* | 3.79 | 45.17 | 76.66 | 31.49 | 49.73 | FM | Zamora-Gutierrez et al. 2016 |
| Chiroptera | Yangochiroptera | Vespertilionidae | *Rhogeessa parvula* | 3.27 | 42.8 | 83.35 | 40.55 | 51.06 | FM | Zamora-Gutierrez et al. 2016 |
| Chiroptera | Yangochiroptera | Vespertilionidae | *Scotophilus heathii* | 2.4 | 60.12 | 37.65 | 22.47 | 41.2 | FM | Luo et al. 2019 |
| Chiroptera | Yangochiroptera | Vespertilionidae | *Scotophilus kuhlii* | 3.2 | 41 | 117.4 | 76.4 | 52.8 | FM | Raghuram et al. 2014 |
| Chiroptera | Yangochiroptera | Vespertilionidae | *Tylonycteris robustula* | 3.3 | 41 | 93.75 | 52.75 | 51.03 | FM | Luo et al. 2019 |
| Chiroptera | Yangochiroptera | Vespertilionidae | *Vespertilio sinensis* | 6.2 | 21.8 | 48.1 | 26.3 | 24.2 | FM | Luo et al. 2019 |
| Chiroptera | Yinochiroptera | Hipposideridae | *Anthops ornatus* | 8.51 | 58.56 | 74.06 | 10.21 | 66.75 | CF | Collen 2012 |
| Chiroptera | Yinochiroptera | Hipposideridae | *Asellia tridens* | 7.97 | 106.96 | 117.74 | 10.78 | 116.6 | CF | Luo et al. 2019 |
| Chiroptera | Yinochiroptera | Hipposideridae | *Aselliscus stoliczkanus* | 4.7 | 110.8 | 127.9 | 17.1 | 127.5 | CF | Luo et al. 2019 |
| Chiroptera | Yinochiroptera | Hipposideridae | *Aselliscus tricuspidatus* | 3.31 | 100.1 | 112.99 | 12.81 | 112.82 | CF | Collen 2012 |
| Chiroptera | Yinochiroptera | Hipposideridae | *Coelops frithii* | 11.8 | 112.5 | 173.7 | 61.2 | 132.9 | FM | Ho Y Y et al. 2013 |
| Chiroptera | Yinochiroptera | Hipposideridae | *Hipposideros abae* | 9.26 | 70.74 | 95.32 | 13.98 | 84.86 | CF | Collen 2012 |
| Chiroptera | Yinochiroptera | Hipposideridae | *Hipposideros armiger* | 24.8 | 61.3 | 68.7 | 7.4 | 68.6 | CF | Ho Y Y et al. 2013 |
| Chiroptera | Yinochiroptera | Hipposideridae | *Hipposideros coronatus* | 8.03 | 82.11 | 91.36 | 10.46 | 90.11 | CF | Collen 2012 |
| Chiroptera | Yinochiroptera | Hipposideridae | *Hipposideros coxi* | 8.68 | 73.41 | 83.56 | 9.35 | 80.96 | CF | Collen 2012 |
| Chiroptera | Yinochiroptera | Hipposideridae | *Hipposideros demissus* | 8.65 | 73.7 | 83.83 | 9.39 | 81.29 | CF | Collen 2012 |
| Chiroptera | Yinochiroptera | Hipposideridae | *Hipposideros diadema* | 11.12 | 51.27 | 60.19 | 8.89 | 59.98 | CF | Collen 2012 |
| Chiroptera | Yinochiroptera | Hipposideridae | *Hipposideros dinops* | 11.55 | 41.68 | 51.45 | 6.46 | 48.28 | CF | Collen 2012 |
| Chiroptera | Yinochiroptera | Hipposideridae | *Hipposideros grandis* | 8.65 | 73.7 | 83.83 | 9.39 | 81.29 | CF | Collen 2012 |
| Chiroptera | Yinochiroptera | Hipposideridae | *Hipposideros lankadiva* | 9.17 | 60.16 | 74.32 | 10.6 | 69.34 | CF | Collen 2012 |
| Chiroptera | Yinochiroptera | Hipposideridae | *Hipposideros lekaguli* | 9.6 | 45.84 | 50.78 | 4.91 | 50.71 | CF | Collen 2012 |
| Chiroptera | Yinochiroptera | Hipposideridae | *Hipposideros lylei* | 9.65 | 58.52 | 67.34 | 8.85 | 67.27 | CF | Collen 2012 |
| Chiroptera | Yinochiroptera | Hipposideridae | *Hipposideros pelingensis* | 8.65 | 73.7 | 83.83 | 9.39 | 81.29 | CF | Collen 2012 |

| | | | | | | | | | | |
|---|---|---|---|---|---|---|---|---|---|---|
| Chiroptera | Yinochiroptera | Hipposideridae | *Hipposideros pratti* | 8.01 | 53.45 | 60.83 | 7.35 | 60.77 | CF | Collen 2012 |
| Chiroptera | Yinochiroptera | Hipposideridae | *Hipposideros scutinares* | 8.65 | 73.7 | 83.83 | 9.39 | 81.29 | CF | Collen 2012 |
| Chiroptera | Yinochiroptera | Hipposideridae | *Macronycteris commersoni* | 14.2 | 58.3 | 73.8 | 15.5 | 64 | CF | Hending et al. 2021 |
| Chiroptera | Yinochiroptera | Hipposideridae | *Rhinonicteris aurantia* | 12 | 100 | 115 | 15 | 114 | CF | Collen 2012 |
| Chiroptera | Yinochiroptera | Megadermatidae | *Megaderma lyra* | 2.6 | 32.9 | 56.4 | 23.5 | 42.5 | FM | Luo et al. 2019 |
| Chiroptera | Yinochiroptera | Megadermatidae | *Megaderma spasma* | 2.06 | 38.87 | 99.79 | 60.92 | 55.9 | FM | Luo et al. 2019 |
| Chiroptera | Yinochiroptera | Pteropodidae | *Rousettus aegyptiacus* | 0.41 | 12 | 70 | 58 | 30 | click | Smarsh etal. 2021 |
| Chiroptera | Yinochiroptera | Pteropodidae | *Rousettus amplexicaudatus* | 0.9 | 13.5 | 65 | 51.5 | 32 | click | Novick 1958; Roberts 1975 |
| Chiroptera | Yinochiroptera | Rhinolophidae | *Rhinolophus acuminatus* | 48.86 | 80.97 | 89.61 | 8.63 | 89.56 | CF | Collen 2012 |
| Chiroptera | Yinochiroptera | Rhinolophidae | *Rhinolophus affinis* | 43.2 | 56.6 | 56.9 | 15.78 | 71.1 | CF | Luo et al. 2019 |
| Chiroptera | Yinochiroptera | Rhinolophidae | *Rhinolophus alcyone* | 35.41 | 72.53 | 91.45 | 15.39 | 87 | CF | Collen 2012 |
| Chiroptera | Yinochiroptera | Rhinolophidae | *Rhinolophus arcuatus* | 35.34 | 57.28 | 69.76 | 10 | 66.5 | CF | Collen 2012 |
| Chiroptera | Yinochiroptera | Rhinolophidae | *Rhinolophus blasii* | 44.1 | 54.33 | 58.31 | 4 | 60.5 | CF | Collen 2012 |
| Chiroptera | Yinochiroptera | Rhinolophidae | *Rhinolophus borneensis* | 31.94 | 69.97 | 83.8 | 12.81 | 81.8 | CF | Collen 2012 |
| Chiroptera | Yinochiroptera | Rhinolophidae | *Rhinolophus capensis* | 42.67 | 71.91 | 84.42 | 12.5 | 84.36 | CF | Collen 2012 |
| Chiroptera | Yinochiroptera | Rhinolophidae | *Rhinolophus celebensis* | 27.74 | 64.91 | 79.35 | 11.76 | 75.04 | CF | Collen 2012 |
| Chiroptera | Yinochiroptera | Rhinolophidae | *Rhinolophus clivosus* | 54.48 | 78.81 | 78.92 | 12.5 | 85.18 | CF | Luo et al. 2019 |
| Chiroptera | Yinochiroptera | Rhinolophidae | *Rhinolophus coelophyllus* | 35.01 | 66.29 | 78.77 | 12.5 | 78.46 | CF | Collen 2012 |
| Chiroptera | Yinochiroptera | Rhinolophidae | *Rhinolophus cornutus* | 43.58 | 92.67 | 106.05 | 13.35 | 105.97 | CF | Collen 2012 |
| Chiroptera | Yinochiroptera | Rhinolophidae | *Rhinolophus creaghi* | 34.64 | 57.97 | 70.95 | 10.53 | 68 | CF | Collen 2012 |
| Chiroptera | Yinochiroptera | Rhinolophidae | *Rhinolophus darlingi* | 36.05 | 73.24 | 86.29 | 13.04 | 86.01 | CF | Collen 2012 |
| Chiroptera | Yinochiroptera | Rhinolophidae | *Rhinolophus denti* | 22.28 | 90.72 | 110.71 | 19.98 | 110.6 | CF | Collen 2012 |
| Chiroptera | Yinochiroptera | Rhinolophidae | *Rhinolophus eloquens* | 38.28 | 36.6 | 45.79 | 6.71 | 43.29 | CF | Collen 2012 |
| Chiroptera | Yinochiroptera | Rhinolophidae | *Rhinolophus euryale* | 21.82 | 91.24 | 106.91 | 15.58 | 105.67 | CF | Collen 2012 |
| Chiroptera | Yinochiroptera | Rhinolophidae | *Rhinolophus euryotis* | 50.82 | 44.92 | 52.03 | 7.1 | 51.99 | CF | Collen 2012 |
| Chiroptera | Yinochiroptera | Rhinolophidae | *Rhinolophus ferrumequinum* | 48.09 | 69.81 | 82.34 | 12.55 | 82.18 | CF | Collen 2012 |
| Chiroptera | Yinochiroptera | Rhinolophidae | *Rhinolophus formosae* | 29.2 | 60.28 | 74.53 | 10.98 | 69.97 | CF | Collen 2012 |
| Chiroptera | Yinochiroptera | Rhinolophidae | *Rhinolophus fumigatus* | 41.27 | 46.57 | 53.28 | 6.64 | 53.24 | CF | Collen 2012 |
| Chiroptera | Yinochiroptera | Rhinolophidae | *Rhinolophus hipposideros* | 40.83 | 94.59 | 109.47 | 14.83 | 109.32 | CF | Collen 2012 |
| Chiroptera | Yinochiroptera | Rhinolophidae | *Rhinolophus inops* | 29.37 | 59.74 | 73.93 | 10.88 | 69.34 | CF | Collen 2012 |
| Chiroptera | Yinochiroptera | Rhinolophidae | *Rhinolophus landeri* | 41.85 | 86.25 | 105.46 | 19.2 | 105.41 | CF | Collen 2012 |
| Chiroptera | Yinochiroptera | Rhinolophidae | *Rhinolophus luctus* | 64.97 | 29.65 | 31.11 | 1.46 | 31.07 | CF | Collen 2012 |
| Chiroptera | Yinochiroptera | Rhinolophidae | *Rhinolophus maclaudi* | 42.52 | 37.98 | 46.85 | 6.34 | 44.93 | CF | Collen 2012 |
| Chiroptera | Yinochiroptera | Rhinolophidae | *Rhinolophus macrotis* | 28.47 | 40.58 | 47 | 6.44 | 46.95 | CF | Collen 2012 |
| Chiroptera | Yinochiroptera | Rhinolophidae | *Rhinolophus malayanus* | 32.04 | 71.68 | 85.62 | 13.95 | 85.55 | CF | Collen 2012 |

| | | | | | | | | | | |
|---|---|---|---|---|---|---|---|---|---|---|
| Chiroptera | Yinochiroptera | Rhinolophidae | *Rhinolophus marshalli* | 49.45 | 38.36 | 45.55 | 4.82 | 43 | CF | Collen 2012 |
| Chiroptera | Yinochiroptera | Rhinolophidae | *Rhinolophus megaphyllus* | 56.06 | 61.29 | 68.85 | 7.56 | 68.8 | CF | Collen 2012 |
| Chiroptera | Yinochiroptera | Rhinolophidae | *Rhinolophus mehelyi* | 35.9 | 97.42 | 89.93 | 5 | 110 | CF | Collen 2012 |
| Chiroptera | Yinochiroptera | Rhinolophidae | *Rhinolophus monoceros* | 29.2 | 93.6 | 108.7 | 15.1 | 108.2 | CF | Ho Y Y et al. 2013 |
| Chiroptera | Yinochiroptera | Rhinolophidae | *Rhinolophus osgoodi* | 28.67 | 61.87 | 76.16 | 11.24 | 71.66 | CF | Collen 2012 |
| Chiroptera | Yinochiroptera | Rhinolophidae | *Rhinolophus paradoxolophus* | 48.76 | 38.71 | 46.23 | 5.07 | 44 | CF | Collen 2012 |
| Chiroptera | Yinochiroptera | Rhinolophidae | *Rhinolophus pearsonii* | 42.3 | 51.42 | 56.72 | 5.33 | 56.68 | CF | Collen 2012 |
| Chiroptera | Yinochiroptera | Rhinolophidae | *Rhinolophus philippinensis* | 81.84 | 35.1 | 39.85 | 4.71 | 39.78 | CF | Collen 2012 |
| Chiroptera | Yinochiroptera | Rhinolophidae | *Rhinolophus pusillus* | 36.08 | 97.72 | 109.93 | 12.18 | 109.85 | CF | Collen 2012 |
| Chiroptera | Yinochiroptera | Rhinolophidae | *Rhinolophus rex* | 43.52 | 22.06 | 26.04 | 3.99 | 26.01 | CF | Collen 2012 |
| Chiroptera | Yinochiroptera | Rhinolophidae | *Rhinolophus robinsoni* | 26.15 | 70.67 | 85.08 | 12.69 | 81.37 | CF | Collen 2012 |
| Chiroptera | Yinochiroptera | Rhinolophidae | *Rhinolophus rufus* | 29.2 | 60.28 | 74.53 | 10.98 | 69.97 | CF | Collen 2012 |
| Chiroptera | Yinochiroptera | Rhinolophidae | *Rhinolophus ruwenzorii* | 39.73 | 39.57 | 48.92 | 6.76 | 46.57 | CF | Collen 2012 |
| Chiroptera | Yinochiroptera | Rhinolophidae | *Rhinolophus shameli* | 34.87 | 61.98 | 69.65 | 7.66 | 69.59 | CF | Collen 2012 |
| Chiroptera | Yinochiroptera | Rhinolophidae | *Rhinolophus siamensis* | 30.85 | 55.92 | 69.69 | 10.09 | 65 | CF | Collen 2012 |
| Chiroptera | Yinochiroptera | Rhinolophidae | *Rhinolophus simulator* | 29.37 | 66.2 | 80.76 | 14.59 | 80.71 | CF | Collen 2012 |
| Chiroptera | Yinochiroptera | Rhinolophidae | *Rhinolophus sinicus* | 32.33 | 71.74 | 86.84 | 13.46 | 84 | CF | Collen 2012 |
| Chiroptera | Yinochiroptera | Rhinolophidae | *Rhinolophus stheno* | 26.53 | 79.48 | 93.99 | 14.52 | 93.89 | CF | Collen 2012 |
| Chiroptera | Yinochiroptera | Rhinolophidae | *Rhinolophus subrufus* | 42.73 | 44.08 | 54.08 | 7.27 | 51 | CF | Collen 2012 |
| Chiroptera | Yinochiroptera | Rhinolophidae | *Rhinolophus swinnyi* | 22.3 | 88.57 | 107.32 | 18.78 | 104.36 | CF | Collen 2012 |
| Chiroptera | Yinochiroptera | Rhinolophidae | *Rhinolophus thomasi* | 33.89 | 74.82 | 89.61 | 14.8 | 89.53 | CF | Collen 2012 |
| Chiroptera | Yinochiroptera | Rhinolophidae | *Rhinolophus trifoliatus* | 19.51 | 44.24 | 52.56 | 8.21 | 52.41 | CF | Collen 2012 |
| Chiroptera | Yinochiroptera | Rhinolophidae | *Rhinolophus virgo* | 38.44 | 59.86 | 70.49 | 9.34 | 67.49 | CF | Collen 2012 |
| Chiroptera | Yinochiroptera | Rhinonycteridae | *Cloeotis percivali* | 2.67 | 188.91 | 209.38 | 20.74 | 209.31 | CF | Collen 2012 |
| Chiroptera | Yinochiroptera | Rhinonycteridae | *Triaenops persicus* | 8.5 | 73.33 | 86.23 | 10.83 | 83 | CF | Collen 2012 |
| Chiroptera | Yinochiroptera | Rhinonycteridae | *Triaenops rufus* | 8.43 | 35.82 | 43.27 | 7.48 | 39.82 | CF | Collen 2012 |
| Chiroptera | Yinochiroptera | Rhinopomatidae | *Rhinopoma hardwickii* | 8.57 | 32.72 | 35.85 | 3.13 | 33.99 | FM | Luo et al. 2019 |
| Dermoptera | NA | Cynocephalidae | *Galeopterus variegatus* | 46.9 | 34.8 | 39.7 | 4.8 | 38.1 | non-echolocation | Miard et al. 2018 |
| Eulipotyphla | NA | Soricidae | *Sorex palustris* | 7 | 30 | 60 | 30 | 41 | click | Gould, Negus and Novick, 1964 |
| Eulipotyphla | NA | Soricidae | *Blarina brevicauda* | 0.6 | 29 | 55 | 26 | 42 | click | Tomasi, 1979 |
| Eulipotyphla | NA | Soricidae | *Sorex minutus* | 11.2 | 6.45 | 7.18 | 0.73 | 6.77 | non-echolocation | Zsebők et al. 2015 |
| Eulipotyphla | NA | Soricidae | *Neomys anomalus* | 15 | 5.58 | 6.2 | 0.62 | 5.84 | non-echolocation | Zsebők et al. 2015 |
| Eulipotyphla | NA | Soricidae | *Crocidura leucodon* | 106.7 | 8.50 | 11.3 | 2.8 | 10.6 | non-echolocation | Simeonovska-Nikolova 2004 |
| Eulipotyphla | NA | Soricidae | *Crocidura russula* | 13.1 | 5.15 | 5.93 | 0.78 | 5.47 | non-echolocation | Zsebők et al. 2015 |
| Eulipotyphla | NA | Soricidae | *Suncus murinus* | 8.8 | 4.17 | 9.28 | 5.11 | 4.97 | non-echolocation | Schneiderová 2014 |
| Eulipotyphla | NA | Solenodontidae | *Solenodon paradoxus* | 0.8 | 9.90 | 31 | 21.1 | 16 | non-echolocation | Eisenberg 1966 |

| | | | | | | | | | | |
|---|---|---|---|---|---|---|---|---|---|---|
| Rodentia | Myomorpha | Cricetidae | *Microtus hartingi* | 31 | 2.6 | 25.6 | 23 | 12.1 | non-echolocation | Rutovskaya 2018 |
| Rodentia | Myomorpha | Cricetidae | *Microtus socialis* | 167 | 1.8 | 8.1 | 6.3 | 5.1 | non-echolocation | Rutovskaya 2018 |
| Rodentia | Myomorpha | Muridae | *Mus musculus* | 29 | 65 | 100 | 35 | 77.6 | non-echolocation | Grimsley et al. 2011 |
| Rodentia | Myomorpha | Muridae | *Pachyuromys duprasi* | 22 | 51.1 | 66.8 | 6.5 | 60 | non-echolocation | Zaytseva et al. 2019 |
| Rodentia | Myomorpha | Platacanthomyidae | *Typhlomys cinereus* | 1.77 | 34.8 | 118.6 | 83.7 | 98.1 | FM | our data |
| Rodentia | Sciuromorpha | Sciuridae | *Glaucomys oregonensis* | 173.67 | 13.62 | 28.72 | 15.1 | 22.17 | non-echolocation | Farwell etal. 2024 |

**Table S3. Performance of *Uropsilus* species under different experimental treatments and conditions.**

| Test groups | Treatment | Experiment apparatus | Species | Sample # | Percentage of exploration time in the left of monitored | Percentage of exploration time in monitored sector (%) | Percentage of exploration time in the opposite sector sector (%) | # of ultrasonic emissions per sec in monitored sector |
|---|---|---|---|---|---|---|---|---|
| Small disks | None | Disc-small discs | *U.gracilis* | UGR01 | 12.89 ± 0.49 | 12.28 ± 0.43 | 13.64 ± 2.22 | 24.21 ± 6.41 |
| | | | | UGR02 | 11.16 ± 1.27 | 11.06 ± 1.14 | 15.11 ± 3.34 | 14.81 ± 1.37 |
| | | | | UGR03 | 12.05 ± 0.98 | 13.04 ± 0.48 | 10.26 ± 2.48 | 16.86 ± 6.76 |
| | | | | UGR04 | 9.7 ± 0.78 | 10.5 ± 1.91 | 13.56 ± 1.79 | 10.9 ± 2.91 |
| | | | | UGR05 | 12.53 ± 2.69 | 10.95 ± 0.88 | 13.29 ± 1.58 | 15.13 ± 6.6 |
| | | | | UGR06 | 14.4 ± 1.91 | 10.79 ± 1.79 | 12 ± 0.81 | 21.74 ± 5.96 |
| | | | | UGR07 | 12.27 ± 1.54 | 12.49 ± 1.14 | 12.03 ± 1.06 | 18.95 ± 4.19 |
| | | | | UGR08 | 11.58 ± 2.15 | 12.39 ± 0.21 | 16.97 ± 3.84 | 18.81 ± 3.77 |
| | | | | UGR09 | 20.29 ± 5.64 | 11.26 ± 1.3 | 11.96 ± 2.02 | 22.26 ± 1.81 |
| | | | | UGR10 | 11.46 ± 0.78 | 13.57 ± 1.74 | 12.22 ± 1.62 | 24.8 ± 4.65 |
| | | | | UGR11 | 11.59 ± 1.73 | 12.75 ± 0.32 | 11.66 ± 0.44 | 20.43 ± 4.42 |
| | | | | UGR12 | 12.58 ± 1.68 | 12.67 ± 1.29 | 9.62 ± 0.9 | 24.46 ± 2.62 |
| | | | | UGR13 | 15.16 ± 1.78 | 13.34 ± 0.71 | 11.09 ± 0.9 | 25.4 ± 5.45 |
| | | | | UGR14 | 14.46 ± 1.96 | 11.78 ± 1.53 | 12.74 ± 0.84 | 14.11 ± 1.84 |
| | | | | UGR15 | 11.76 ± 1.39 | 11.52 ± 1.32 | 9.65 ± 2.39 | 16.25 ± 4.48 |
| | | | | UGR16 | 16.03 ± 6.5 | 10.91 ± 0.56 | 9.14 ± 2.12 | 9.14 ± 2.49 |
| | | | *U.investigator* | UIN01 | 14.46 ± 1.99 | 13.85 ± 2.53 | 15.33 ± 4.76 | 14.8 ± 7.74 |
| | | | | UIN02 | 13.75 ± 2.33 | 14.12 ± 3.34 | 5.86 ± 2.06 | 30.23 ± 12.79 |
| | | | | UIN03 | 11.67 ± 0.7 | 11.77 ± 2.05 | 9.71 ± 1.77 | 24.9 ± 7.97 |
| | | | | UIN04 | 12.69 ± 0.98 | 10.95 ± 0.65 | 14.05 ± 1.02 | 19.81 ± 4.82 |
| | | | | UIN05 | 13.7 ± 4.2 | 14.85 ± 5.15 | 11.23 ± 1.65 | 17.56 ± 11.16 |
| | | | | UIN06 | 12.13 ± 4.18 | 14.26 ± 3.87 | 10.15 ± 3.13 | 26.07 ± 11.58 |
| | | | | UIN07 | 9.38 ± 1.09 | 15.7 ± 2.12 | 13.67 ± 2.29 | 29.79 ± 3.82 |
| | | | *U.nivatus* | UNI01 | 11.06 ± 0.33 | 13.68 ± 0.48 | 13.05 ± 0.92 | 16.2 ± 3.65 |
| | | | | UNI02 | 11.46 ± 1.31 | 10.59 ± 2.01 | 14.05 ± 2.28 | 27.61 ± 5.29 |
| | | | | UNI03 | 10.13 ± 0.89 | 11.25 ± 0.48 | 16.03 ± 2.54 | 26.99 ± 2.85 |
| | | | | UNI04 | 11.59 ± 0.92 | 13.2 ± 0.88 | 12.3 ± 1.02 | 23.13 ± 5.92 |
| | | | | UNI05 | 12.31 ± 1.47 | 13.25 ± 1.11 | 13.1 ± 0.61 | 20.4 ± 7.92 |
| | | | | UNI06 | 13.27 ± 1.89 | 13.92 ± 1.67 | 10.89 ± 2 | 28.19 ± 4.63 |
| | | | | UNI07 | 12.77 ± 1.17 | 12.32 ± 0.27 | 12.46 ± 0.86 | 16.96 ± 7.01 |
| | | | | UNI08 | 14.64 ± 1.73 | 12.94 ± 1.02 | 11.55 ± 1.12 | 41.64 ± 7.04 |
| | | | | UNI09 | 11.36 ± 1.14 | 11.86 ± 1.99 | 13.83 ± 1.67 | 24.75 ± 9.94 |
| | | | | UNI10 | 12.02 ± 1.07 | 12.52 ± 0.8 | 12.28 ± 1.18 | 18.37 ± 1.87 |
| | | | | UNI11 | 11.51 ± 1.74 | 13.09 ± 0.9 | 13.83 ± 1.26 | 18.74 ± 5.79 |
| | | | *U. soricipes* | USO01 | 13.87 ± 3.91 | 10.3 ± 0.64 | 13.96 ± 2.91 | 18.55 ± 2.75 |
| | | | | USO02 | 9.53 ± 2.96 | 13.5 ± 1.66 | 12.21 ± 2.04 | 17.68 ± 1.17 |
| | | | | USO03 | 9.53 ± 1.14 | 12.55 ± 0.96 | 11.15 ± 2.19 | 22.22 ± 7.31 |
| | | | | USO04 | 12.76 ± 1.16 | 10.29 ± 0.68 | 11.87 ± 1.25 | 16.42 ± 1.61 |
| | | | | USO05 | 10.24 ± 1.55 | 11.28 ± 0.69 | 11.84 ± 1.09 | 20.2 ± 3.03 |
| | | | | USO06 | 10.84 ± 1.69 | 11.24 ± 0.98 | 10.81 ± 2.36 | 22.91 ± 1.88 |
| | | | | USO07 | 11.06 ± 1.48 | 11.62 ± 0.61 | 11.34 ± 1.67 | 16.85 ± 3.21 |
| | | | | USO08 | 14.27 ± 0.94 | 13.46 ± 1.02 | 11.34 ± 1.19 | 15.24 ± 2.75 |
| | | | | USO09 | 13.65 ± 2.46 | 13.3 ± 0.95 | 12.36 ± 1.76 | 19.28 ± 3.64 |
| | | | | USO10 | 13.08 ± 3.19 | 11.9 ± 1.2 | 15.42 ± 3.57 | 22.34 ± 4.32 |
| | | | | USO11 | 12.52 ± 4.8 | 10.51 ± 0.36 | 13.24 ± 2.34 | 16.14 ± 2.11 |
| | | | | USO12 | 17.34 ± 3.98 | 13.21 ± 1.63 | 11.52 ± 2.59 | 18.45 ± 3.27 |
| | | | | USO13 | 14.35 ± 1.71 | 14.24 ± 0.76 | 9.99 ± 0.9 | 19.71 ± 4.15 |
| | | | | USO14 | 10.61 ± 2.8 | 10.57 ± 1.3 | 9.97 ± 0.91 | 25.22 ± 7.1 |
| Platform | None | Disc-platform | *U.gracilis* | UGR01 | 12.24 ± 2.71 | 33.87 ± 7.29 | 6.57 ± 2.28 | 27.39 ± 7.54 |
| | | | | UGR02 | 8.92 ± 1.61 | 31.07 ± 3.38 | 10.43 ± 3.84 | 23.71 ± 5.71 |
| | | | | UGR03 | 10.7 ± 1.56 | 33.56 ± 7.22 | 7.86 ± 1.78 | 16.35 ± 5.18 |
| | | | | UGR04 | 15.29 ± 2.65 | 33.18 ± 5.96 | 9.5 ± 4.44 | 23.41 ± 9.57 |
| | | | | UGR05 | 11.61 ± 2.78 | 37.87 ± 5.28 | 8.47 ± 1.39 | 35.9 ± 6.42 |
| | | | | UGR06 | 9.86 ± 2.07 | 29.16 ± 2.95 | 13.87 ± 4.01 | 21.24 ± 4.32 |
| | | | | UGR07 | 11.42 ± 1.28 | 27.82 ± 3.64 | 8.17 ± 1.48 | 32.66 ± 7.02 |
| | | | | UGR08 | 8.74 ± 2.77 | 26.4 ± 6.87 | 6.24 ± 2.38 | 25.23 ± 6.12 |
| | | | | UGR09 | 10.69 ± 0.96 | 24 ± 3.06 | 11.99 ± 2.96 | 39.76 ± 6.28 |
| | | | | UGR10 | 11.3 ± 2.49 | 32.44 ± 6.92 | 11.47 ± 5.59 | 24.78 ± 4.44 |

| | | | | | | | | |
|---|---|---|---|---|---|---|---|---|
| Platform | None | Disc-platform | *U.gracilis* | UGR11 | 14.01 ± 3.17 | 28.95 ± 3.15 | 8.46 ± 1.83 | 31.66 ± 7.57 |
| | | | | UGR12 | 14.78 ± 1.21 | 20.54 ± 1.51 | 5.62 ± 1.68 | 21.18 ± 3.94 |
| | | | | UGR13 | 15.98 ± 6.51 | 31.93 ± 4 | 4.82 ± 1.19 | 17.67 ± 4.73 |
| | | | | UGR14 | 13.48 ± 2.28 | 32.28 ± 5.68 | 7.58 ± 1.8 | 27.51 ± 7.39 |
| | | | | UGR15 | 7.37 ± 1.04 | 37.17 ± 4.46 | 7.34 ± 2.66 | 15.91 ± 4.85 |
| | | | | UGR16 | 12.25 ± 0.36 | 31.78 ± 8.31 | 9.97 ± 1.71 | 29.42 ± 8.85 |
| | | | *U.investigator* | UIN01 | 9.97 ± 2.01 | 28.58 ± 4.73 | 6.66 ± 2.09 | 42.87 ± 6.6 |
| | | | | UIN02 | 6.52 ± 5.29 | 23.02 ± 4.79 | 4.55 ± 2.63 | 37.92 ± 10.61 |
| | | | | UIN03 | 11.77 ± 0.86 | 21.49 ± 4.37 | 10.97 ± 4.49 | 47.67 ± 13.71 |
| | | | | UIN04 | 20.52 ± 3.14 | 22.83 ± 3.18 | 9.94 ± 3 | 49 ± 5.38 |
| | | | | UIN05 | 18.74 ± 4.5 | 25.15 ± 7.85 | 8.15 ± 3.03 | 41.93 ± 8.93 |
| | | | | UIN06 | 13.32 ± 9.73 | 24.1 ± 5.49 | 2.76 ± 1.83 | 45.3 ± 5.81 |
| | | | | UIN07 | 5.49 ± 2.36 | 22.08 ± 2.09 | 5.02 ± 2.27 | 47.58 ± 7.71 |
| | | | *U.nivatus* | UNI01 | 17.09 ± 4.69 | 26.07 ± 2.01 | 7.05 ± 1.82 | 31.48 ± 9.52 |
| | | | | UNI02 | 15.32 ± 2.24 | 23.29 ± 2.37 | 6.28 ± 2.78 | 36.52 ± 11.13 |
| | | | | UNI03 | 14.93 ± 1.65 | 24.81 ± 1.5 | 6.36 ± 1.57 | 39.94 ± 4.83 |
| | | | | UNI04 | 14.08 ± 2.01 | 23.16 ± 1.75 | 12.2 ± 2.57 | 29.58 ± 1.23 |
| | | | | UNI05 | 11.95 ± 0.57 | 21.85 ± 1.53 | 12.35 ± 1.48 | 37.8 ± 5.88 |
| | | | | UNI06 | 9.99 ± 2.24 | 22.89 ± 1.31 | 13.73 ± 3.58 | 33.48 ± 5.52 |
| | | | | UNI07 | 12.55 ± 1.73 | 21.86 ± 0.86 | 9.53 ± 1.54 | 27.7 ± 5.9 |
| | | | | UNI08 | 11.73 ± 0.55 | 23.92 ± 1.56 | 8.13 ± 0.44 | 40.74 ± 6.65 |
| | | | | UNI09 | 10.36 ± 1.79 | 35.69 ± 4.96 | 5.4 ± 2.05 | 32.43 ± 10.73 |
| | | | | UNI10 | 13.6 ± 2.02 | 30.2 ± 6.77 | 11.19 ± 5.93 | 35.05 ± 2.22 |
| | | | | UNI11 | 15.93 ± 0.92 | 28.81 ± 4.5 | 9.07 ± 1.58 | 35.43 ± 5.94 |
| | | | *U. soricipes* | USO01 | 13 ± 1.12 | 28.8 ± 2.74 | 5.98 ± 1.87 | 28.7 ± 2.7 |
| | | | | USO02 | 17.72 ± 2.75 | 27.65 ± 2.39 | 6.42 ± 1.5 | 26.54 ± 3.05 |
| | | | | USO03 | 10.39 ± 1.89 | 33.74 ± 4.55 | 7.81 ± 1.47 | 29.89 ± 2.78 |
| | | | | USO04 | 14.22 ± 2.65 | 25.5 ± 3.82 | 5.79 ± 1.73 | 22.57 ± 4.5 |
| | | | | USO05 | 15.34 ± 0.82 | 26.86 ± 2.16 | 8.27 ± 1.65 | 23.29 ± 1.96 |
| | | | | USO06 | 19.33 ± 1.88 | 26.61 ± 2.04 | 7.36 ± 2.37 | 29.42 ± 2.65 |
| | | | | USO07 | 23.15 ± 8.36 | 28.73 ± 2 | 5.16 ± 1.48 | 36.33 ± 3.23 |
| | | | | USO08 | 17.56 ± 3.17 | 34.4 ± 7.11 | 7.4 ± 2.63 | 26.05 ± 5.91 |
| | | | | USO09 | 14.55 ± 1.54 | 26.36 ± 2.24 | 9.3 ± 1.49 | 30.01 ± 6.21 |
| | | | | USO10 | 15.08 ± 3.43 | 26.6 ± 2.35 | 9.11 ± 1.03 | 20.49 ± 3.81 |
| | | | | USO11 | 16.35 ± 3.36 | 22.84 ± 1.33 | 7.86 ± 1.86 | 32.29 ± 4.25 |
| | | | | USO12 | 13.5 ± 0.71 | 27.41 ± 4.2 | 8.16 ± 0.54 | 25.05 ± 3.31 |
| | | | | USO13 | 11.71 ± 3.49 | 25.82 ± 3.01 | 6.58 ± 1.79 | 33.57 ± 4.39 |
| | | | | USO14 | 12.88 ± 1.69 | 27.92 ± 2.22 | 9.54 ± 3.28 | 28.75 ± 6.78 |
| Earplug | Ears plugged | Disc-platform | *U.gracilis* | UGR01 | 13.94 ± 1.46 | 11.26 ± 1.81 | 12.26 ± 1.66 | 10.5 ± 3.74 |
| | | | | UGR02 | 16.3 ± 2.57 | 12.82 ± 1.41 | 12.23 ± 1.99 | 8.07 ± 4.09 |
| | | | | UGR03 | 11.35 ± 4.32 | 13.02 ± 1.97 | 13.99 ± 1.74 | 10.35 ± 3.33 |
| | | | | UGR04 | 11.05 ± 2.09 | 12.65 ± 1.74 | 13.53 ± 2.13 | 7.82 ± 4.58 |
| | | | | UGR05 | 10.31 ± 1.28 | 12.89 ± 1.84 | 10.95 ± 2.41 | 14.9 ± 4.35 |
| | | | | UGR06 | 12.12 ± 2.64 | 14.3 ± 1.11 | 8.3 ± 2.05 | 13.93 ± 3.91 |
| | | | | UGR07 | 11.69 ± 2.33 | 11.5 ± 1.13 | 10.37 ± 0.87 | 23.56 ± 9.73 |
| | | | | UGR08 | 12.89 ± 0.66 | 13.22 ± 1.45 | 10.8 ± 1.31 | 21.54 ± 5.93 |
| | | | | UGR09 | 12.26 ± 1.74 | 12.59 ± 1.25 | 15.5 ± 2.18 | 15.17 ± 1.26 |
| | | | | UGR10 | 15.42 ± 2.42 | 12.42 ± 1 | 13.29 ± 0.99 | 19.9 ± 4.8 |
| | | | | UGR11 | 11.68 ± 1.92 | 12 ± 1.34 | 10.29 ± 0.32 | 13.31 ± 7.76 |
| | | | | UGR12 | 15.06 ± 1.84 | 12.87 ± 0.67 | 13.56 ± 1.09 | 11.65 ± 4.36 |
| | | | | UGR13 | 18.7 ± 3.25 | 14.36 ± 0.75 | 11.55 ± 1.18 | 19.83 ± 6.71 |
| | | | | UGR14 | 9.7 ± 3.5 | 12.82 ± 1.68 | 14.51 ± 1.29 | 9.91 ± 4.91 |
| | | | | UGR15 | 9.23 ± 0.17 | 9.88 ± 0.54 | 12.26 ± 1.18 | 24.36 ± 8.1 |
| | | | | UGR16 | 11.13 ± 0.39 | 11.69 ± 0.87 | 18.03 ± 3.05 | 10.44 ± 5.91 |
| | | | *U.investigator* | UIN01 | 10.11 ± 3.64 | 9.75 ± 3.2 | 16.19 ± 3.88 | 7.15 ± 3.14 |
| | | | | UIN02 | 14.14 ± 2.26 | 12.99 ± 3.4 | 8.87 ± 1.66 | 25.71 ± 6.01 |
| | | | | UIN03 | 16.25 ± 0.78 | 13.51 ± 1.41 | 9.08 ± 1.22 | 37.41 ± 5.72 |
| | | | | UIN04 | 10.48 ± 3.63 | 11.14 ± 0.61 | 14.4 ± 3.24 | 29.26 ± 2.94 |
| | | | | UIN05 | 14.7 ± 1.59 | 15.73 ± 2.27 | 11.65 ± 1.6 | 18.5 ± 5.63 |
| | | | | UIN06 | 13.81 ± 2.41 | 14.44 ± 2.73 | 7.85 ± 1.01 | 22.44 ± 5.65 |
| | | | | UIN07 | 8.52 ± 1.46 | 11.49 ± 4.63 | 13.87 ± 2.08 | 20.04 ± 4.38 |
| | | | *U.nivatus* | UNI01 | 12.29 ± 1.2 | 14.01 ± 1.86 | 11.73 ± 0.43 | 8.79 ± 1.49 |
| | | | | UNI02 | 11.09 ± 1.97 | 14.41 ± 2.41 | 13.23 ± 1.67 | 28.91 ± 12.82 |
| | | | | UNI03 | 13.91 ± 2.08 | 13.51 ± 1.79 | 12.91 ± 1.49 | 12.59 ± 3.39 |
| | | | | UNI04 | 10.91 ± 0.83 | 12.69 ± 1.26 | 14.47 ± 1.99 | 23.28 ± 1.27 |

| | | | | | | | | |
|---|---|---|---|---|---|---|---|---|
| Earplug | Ears plugged | Disc-platform | *U.nivatus* | UNI05 | 12.26 ± 2.97 | 10.63 ± 1.88 | 14.93 ± 1.86 | 13.61 ± 3.29 |
| | | | | UNI06 | 15.23 ± 0.96 | 13.4 ± 1.26 | 14.63 ± 1.62 | 13.3 ± 2.72 |
| | | | | UNI07 | 12.8 ± 1 | 11.98 ± 0.75 | 15.45 ± 1.2 | 18.68 ± 6.03 |
| | | | | UNI08 | 14.72 ± 1.84 | 11.17 ± 0.65 | 14.08 ± 0.76 | 13.99 ± 2.54 |
| | | | | UNI09 | 7.91 ± 2.77 | 13.66 ± 0.31 | 19.21 ± 4.35 | 7.95 ± 1.83 |
| | | | | UNI10 | 11.52 ± 0.63 | 12.62 ± 2.3 | 14.93 ± 1.16 | 10.47 ± 1.67 |
| | | | | UNI11 | 14.12 ± 2.48 | 13.12 ± 1.97 | 11.22 ± 1.2 | 18.71 ± 4.76 |
| | | | *U. soricipes* | USO01 | 19.21 ± 3.71 | 10.35 ± 0.65 | 10.56 ± 2.91 | 19.37 ± 4.93 |
| | | | | USO02 | 15.23 ± 3.26 | 12.71 ± 1.1 | 10.79 ± 5.21 | 20.31 ± 5.36 |
| | | | | USO03 | 9.95 ± 1.78 | 11.48 ± 0.75 | 15.44 ± 2.97 | 14.02 ± 2.34 |
| | | | | USO04 | 10.7 ± 1.47 | 13.82 ± 0.36 | 12.59 ± 2.59 | 22.53 ± 1.55 |
| | | | | USO05 | 15.63 ± 3.81 | 11.48 ± 1.2 | 12.48 ± 1.42 | 13.92 ± 3.55 |
| | | | | USO06 | 11.05 ± 4.15 | 11.68 ± 1.54 | 10.02 ± 1.98 | 18.78 ± 5.55 |
| | | | | USO07 | 11.6 ± 3.19 | 10.89 ± 0.91 | 11.92 ± 2.81 | 19.78 ± 3.48 |
| | | | | USO08 | 12.41 ± 1.6 | 12.68 ± 1.21 | 11.63 ± 1.04 | 15.65 ± 3.97 |
| | | | | USO09 | 14.9 ± 3.64 | 14.74 ± 0.85 | 10.5 ± 1.11 | 15.52 ± 1.96 |
| | | | | USO10 | 13.74 ± 1.42 | 11.16 ± 0.88 | 13.64 ± 1.94 | 19.94 ± 5.89 |
| | | | | USO11 | 18.58 ± 4.2 | 12.19 ± 0.53 | 10.92 ± 2.35 | 18.36 ± 4.67 |
| | | | | USO12 | 10.21 ± 0.82 | 12.55 ± 1.31 | 12.78 ± 2.8 | 17.52 ± 3.96 |
| | | | | USO13 | 12.52 ± 2.24 | 12.26 ± 1.19 | 12.57 ± 1.45 | 16.17 ± 2.01 |
| | | | | USO14 | 9.11 ± 1.67 | 12.18 ± 0.5 | 14.62 ± 2.76 | 18.91 ± 2.96 |
| Sham | Hollow tubes | Disc-platform | *U.gracilis* | UGR01 | 14.6 ± 1.65 | 25.14 ± 1.67 | 13.43 ± 4.79 | 17.85 ± 1.78 |
| | | | | UGR02 | 14.27 ± 1.53 | 27.9 ± 2.81 | 10.9 ± 3.58 | 20.15 ± 4.68 |
| | | | | UGR03 | 10.33 ± 2.36 | 24.11 ± 3.61 | 8.55 ± 0.74 | 17.1 ± 6.67 |
| | | | | UGR04 | 13.53 ± 1.12 | 29.92 ± 5.47 | 6.85 ± 1.87 | 27.64 ± 5.14 |
| | | | | UGR05 | 12.79 ± 0.91 | 39.48 ± 4.21 | 6.92 ± 1.28 | 20.44 ± 2.55 |
| | | | | UGR06 | 11.52 ± 1.46 | 29.51 ± 5.73 | 8.26 ± 2.19 | 22.18 ± 3.88 |
| | | | | UGR07 | 15.54 ± 2.92 | 24.5 ± 5.1 | 7.78 ± 0.49 | 35.69 ± 5.95 |
| | | | | UGR08 | 13.43 ± 5.44 | 32.56 ± 6.51 | 4.16 ± 1.24 | 27.22 ± 1.99 |
| | | | | UGR09 | 13.38 ± 3.61 | 27.55 ± 4.81 | 12.11 ± 4.26 | 23.5 ± 8.5 |
| | | | | UGR10 | 13.16 ± 2.41 | 25.89 ± 4 | 11.47 ± 1.41 | 28.18 ± 3.51 |
| | | | | UGR11 | 12.59 ± 1.97 | 28.49 ± 4.81 | 11.47 ± 1.82 | 27.58 ± 1.37 |
| | | | | UGR12 | 12.79 ± 0.86 | 19.01 ± 1.38 | 17.34 ± 4.78 | 31.73 ± 4.39 |
| | | | | UGR13 | 9.86 ± 2.25 | 29.38 ± 5.07 | 10.08 ± 1.67 | 19.58 ± 7.52 |
| | | | | UGR14 | 14.3 ± 3 | 29.94 ± 5.62 | 8.31 ± 1.6 | 17.78 ± 2.99 |
| | | | | UGR15 | 12.47 ± 1.66 | 43.76 ± 4.72 | 6.38 ± 2.12 | 27.29 ± 4.71 |
| | | | | UGR16 | 13.06 ± 3.07 | 24.84 ± 1.69 | 13.94 ± 4.13 | 22.64 ± 4.67 |
| | | | *U.investigator* | UIN01 | 12.5 ± 4.38 | 17.85 ± 3.79 | 14.71 ± 5.95 | 46.18 ± 8.67 |
| | | | | UIN02 | 17.15 ± 7.91 | 18.04 ± 3.17 | 6.65 ± 2.32 | 41.85 ± 12.41 |
| | | | | UIN03 | 11.82 ± 2.3 | 19.86 ± 3.16 | 10.14 ± 1.75 | 34.83 ± 3.86 |
| | | | | UIN04 | 14.66 ± 2.66 | 21.81 ± 3.6 | 6.17 ± 2 | 26.24 ± 8.29 |
| | | | | UIN05 | 15.11 ± 2.13 | 31.21 ± 4.5 | 8.83 ± 4.5 | 42.62 ± 11.09 |
| | | | | UIN06 | 12.04 ± 2.06 | 29.59 ± 7.27 | 7.78 ± 2.25 | 39.39 ± 8.75 |
| | | | | UIN07 | 10.01 ± 3.03 | 34.37 ± 9.78 | 4.23 ± 1.34 | 37.81 ± 6.55 |
| | | | *U.nivatus* | UNI01 | 9.58 ± 0.91 | 23.48 ± 3.56 | 10.15 ± 0.95 | 41.69 ± 6.71 |
| | | | | UNI02 | 10.86 ± 3.24 | 27.03 ± 4.78 | 7.93 ± 2.2 | 50.65 ± 6.83 |
| | | | | UNI03 | 17.9 ± 3.6 | 25.69 ± 3.92 | 8.92 ± 3.05 | 34.35 ± 3.72 |
| | | | | UNI04 | 12.66 ± 0.86 | 24.2 ± 4.05 | 10.23 ± 0.85 | 52.74 ± 7.86 |
| | | | | UNI05 | 12.69 ± 2.89 | 24.72 ± 3.85 | 11.5 ± 1.51 | 41.93 ± 3.98 |
| | | | | UNI06 | 14.94 ± 3.81 | 22.94 ± 1.67 | 8.83 ± 0.95 | 33.35 ± 4.63 |
| | | | | UNI07 | 11.88 ± 0.99 | 24.97 ± 1.82 | 8.49 ± 2.22 | 35.09 ± 5.23 |
| | | | | UNI08 | 14.13 ± 1.69 | 33.42 ± 3.52 | 7.24 ± 2.32 | 33.02 ± 9.2 |
| | | | | UNI09 | 9.78 ± 2.81 | 30.44 ± 5.99 | 11.61 ± 6.4 | 43.9 ± 9.41 |
| | | | | UNI10 | 17.1 ± 1.93 | 40.33 ± 5.7 | 3.72 ± 1.35 | 31.04 ± 7.82 |
| | | | | UNI11 | 14.83 ± 2.06 | 34.1 ± 3.38 | 5.35 ± 2.43 | 27.8 ± 8.56 |
| | | | *U. soricipes* | USO01 | 11.89 ± 1.53 | 26.3 ± 2.61 | 10.09 ± 1.8 | 26.91 ± 7.08 |
| | | | | USO02 | 14.48 ± 4.66 | 25.63 ± 1.82 | 6.8 ± 1.8 | 28.25 ± 1.47 |
| | | | | USO03 | 10.82 ± 0.85 | 27.99 ± 4.02 | 8.69 ± 0.5 | 25.29 ± 3.85 |
| | | | | USO04 | 12.84 ± 1.15 | 29.5 ± 4.71 | 6.12 ± 0.54 | 22.87 ± 3.74 |
| | | | | USO05 | 8.82 ± 2.71 | 31.45 ± 6.38 | 10.1 ± 0.93 | 21.41 ± 1.01 |
| | | | | USO06 | 9.11 ± 2.37 | 38.24 ± 5.81 | 7.41 ± 0.93 | 37.21 ± 2.48 |
| | | | | USO07 | 20 ± 3.33 | 32.42 ± 3.28 | 4.03 ± 1.87 | 35.47 ± 3.27 |
| | | | | USO08 | 12.78 ± 4.59 | 30.47 ± 3.13 | 7 ± 2.33 | 31.01 ± 3.34 |
| | | | | USO09 | 14.33 ± 3.12 | 27.55 ± 2.86 | 5.48 ± 1.32 | 23.54 ± 5.34 |
| | | | | USO10 | 13.83 ± 2.39 | 30.46 ± 4.2 | 6.63 ± 1.11 | 25.26 ± 4.78 |

| | | | | | | | | |
|---|---|---|---|---|---|---|---|---|
| Sham | Hollow tubes | Disc-platform | *U. Soricipes* | USO11 | 14.99 ± 1.92 | 28.41 ± 2.09 | 9.93 ± 1.8 | 32.68 ± 4.74 |
| | | | | USO12 | 13.13 ± 2.71 | 23.24 ± 0.86 | 9.03 ± 4.55 | 23.46 ± 1.82 |
| | | | | USO13 | 12.54 ± 1.02 | 28.7 ± 2.44 | 10.39 ± 2.15 | 29.56 ± 0.74 |
| | | | | USO14 | 11.65 ± 1.3 | 30.09 ± 4.39 | 9.53 ± 0.44 | 25.98 ± 2.83 |
| Recovery | None | Disc-platform | *U.gracilis* | UGR01 | 12.81 ± 0.67 | 29.91 ± 2.74 | 9.86 ± 2.36 | 35.72 ± 11.15 |
| | | | | UGR02 | 12.37 ± 0.84 | 28.34 ± 2.23 | 7.25 ± 0.8 | 34.17 ± 6.91 |
| | | | | UGR03 | 11.07 ± 0.83 | 25.71 ± 5.53 | 8.35 ± 2.55 | 21.6 ± 2.32 |
| | | | | UGR04 | 12.19 ± 4.71 | 27.2 ± 5.48 | 13.71 ± 6.13 | 20.47 ± 5.82 |
| | | | | UGR05 | 10.55 ± 1.12 | 45 ± 9.67 | 5.47 ± 0.8 | 21.59 ± 2.7 |
| | | | | UGR06 | 9.81 ± 1.56 | 26.6 ± 5.86 | 11.29 ± 3.52 | 22.39 ± 1.59 |
| | | | | UGR07 | 16.74 ± 2.88 | 36.38 ± 5.78 | 8.32 ± 1.32 | 36.37 ± 8.4 |
| | | | | UGR08 | 10.14 ± 2.48 | 31.02 ± 4.43 | 8.43 ± 1.54 | 44.3 ± 8.22 |
| | | | | UGR09 | 10.73 ± 1.33 | 26.47 ± 2.35 | 9.74 ± 2.3 | 25.29 ± 6.25 |
| | | | | UGR10 | 14.78 ± 2.69 | 24.82 ± 3.9 | 8.09 ± 0.96 | 41.51 ± 5.32 |
| | | | | UGR11 | 12.1 ± 1.96 | 27.79 ± 5.45 | 10.08 ± 1.3 | 27.34 ± 5.17 |
| | | | | UGR12 | 15.71 ± 3.41 | 21.04 ± 3.82 | 13.88 ± 4.06 | 30.28 ± 6.36 |
| | | | | UGR13 | 21.44 ± 4.25 | 37.62 ± 5.37 | 5.13 ± 1.15 | 36.82 ± 7.13 |
| | | | | UGR14 | 13.51 ± 1.78 | 26.97 ± 4.23 | 6.93 ± 0.66 | 36.78 ± 5.62 |
| | | | | UGR15 | 10.45 ± 2.08 | 40.07 ± 5.52 | 7.71 ± 0.81 | 33.76 ± 6.85 |
| | | | | UGR16 | 11.47 ± 1.58 | 27.32 ± 2.98 | 11.35 ± 1.37 | 28.67 ± 4.04 |
| | | | *U.investigator* | UIN01 | 8.91 ± 1.37 | 23.55 ± 3.53 | 10.14 ± 3.35 | 39.98 ± 5.68 |
| | | | | UIN02 | 10.28 ± 2.34 | 24.55 ± 4.57 | 8.33 ± 3.09 | 43.24 ± 13.01 |
| | | | | UIN03 | 16.61 ± 4.51 | 32.93 ± 5.25 | 5.56 ± 1.84 | 30.67 ± 7.72 |
| | | | | UIN04 | 10.64 ± 5.88 | 27.14 ± 8.89 | 8.19 ± 2.93 | 43.65 ± 4.3 |
| | | | | UIN05 | 12.23 ± 3.09 | 18.77 ± 3.46 | 12.45 ± 2.68 | 36.17 ± 5.61 |
| | | | | UIN06 | 9.76 ± 3.98 | 31.27 ± 10.61 | 5.77 ± 2.75 | 37.63 ± 0.95 |
| | | | | UIN07 | 26.48 ± 8.85 | 26.46 ± 6.17 | 12.1 ± 3.54 | 39.79 ± 1.92 |
| | | | *U.nivatus* | UNI01 | 12.21 ± 2.21 | 24.22 ± 2.65 | 10.41 ± 2.77 | 37 ± 3.68 |
| | | | | UNI02 | 12.72 ± 3.19 | 28.83 ± 3.47 | 7.18 ± 3.23 | 44.53 ± 12.12 |
| | | | | UNI03 | 13.53 ± 2.83 | 25.14 ± 0.89 | 9 ± 1.78 | 15.67 ± 0.81 |
| | | | | UNI04 | 11.72 ± 1.17 | 20.55 ± 0.44 | 12.09 ± 0.77 | 38.82 ± 6.26 |
| | | | | UNI05 | 11.95 ± 2.26 | 23.85 ± 1.17 | 9.95 ± 0.77 | 33.55 ± 2.2 |
| | | | | UNI06 | 12.46 ± 2.92 | 28.29 ± 2.82 | 10.2 ± 0.36 | 42.57 ± 15.64 |
| | | | | UNI07 | 13.04 ± 2.65 | 27.59 ± 0.21 | 10.51 ± 4.41 | 37.51 ± 5.89 |
| | | | | UNI08 | 12.31 ± 2.06 | 37.43 ± 3.76 | 9.87 ± 3 | 27.68 ± 6.92 |
| | | | | UNI09 | 9.6 ± 0.84 | 32.7 ± 6.36 | 9.8 ± 2.8 | 25.55 ± 3.54 |
| | | | | UNI10 | 10.5 ± 2.56 | 47.78 ± 3.39 | 5.86 ± 2.07 | 37.22 ± 6.93 |
| | | | | UNI11 | 10.35 ± 1.97 | 28.68 ± 4.61 | 10.28 ± 0.28 | 39.06 ± 12.39 |
| | | | *U. soricipes* | USO01 | 12.38 ± 5.1 | 29.83 ± 7.39 | 4.67 ± 1.23 | 37.67 ± 4.15 |
| | | | | USO02 | 13.81 ± 6.11 | 37.5 ± 5.79 | 6.06 ± 4.29 | 32.2 ± 11.51 |
| | | | | USO03 | 12.07 ± 4.14 | 27.42 ± 5.2 | 9.24 ± 2.6 | 23.39 ± 2.36 |
| | | | | USO04 | 13.29 ± 4.83 | 31.05 ± 3.75 | 9.85 ± 3.47 | 36.04 ± 2.33 |
| | | | | USO05 | 11.63 ± 6.43 | 29.47 ± 4.21 | 10.17 ± 6.6 | 39.07 ± 5.04 |
| | | | | USO06 | 8.2 ± 2.14 | 32.48 ± 4.87 | 10.01 ± 3.75 | 33.26 ± 6.45 |
| | | | | USO07 | 10.88 ± 2.18 | 39.62 ± 8.99 | 7.67 ± 1.38 | 25.42 ± 1.99 |
| | | | | USO08 | 6.14 ± 2.17 | 32.3 ± 5.13 | 16.24 ± 3.64 | 27.33 ± 1.02 |
| | | | | USO09 | 11.65 ± 2.85 | 21.75 ± 3.55 | 9.69 ± 1.85 | 28.64 ± 6.58 |
| | | | | USO10 | 14.42 ± 5.49 | 22.18 ± 0.82 | 8.68 ± 1.52 | 25.28 ± 1.9 |
| | | | | USO11 | 11.68 ± 3.14 | 20.18 ± 0.58 | 8.11 ± 1.5 | 22.19 ± 1.01 |
| | | | | USO12 | 15.97 ± 3.72 | 20.88 ± 0.32 | 10.99 ± 1.52 | 39.46 ± 4.64 |
| | | | | USO13 | 11.21 ± 1.85 | 30.96 ± 10.4 | 8.41 ± 3.28 | 31.26 ± 3.9 |
| | | | | USO14 | 13.66 ± 3.1 | 27.89 ± 6.61 | 7.97 ± 2.44 | 26.96 ± 5.87 |

**Table S4. The rate of pulses produced by Asian shrew moles, tongue-click-echolocating and laryngeally echolocating species when their tongue were ligatured.**

| Species | Experiment treatment | Sample # | pluses rate(/S) |
|---|---|---|---|
| *Rousettus leschenaultii* | None (before experiment) | RLE1 | 13.71 |
| | | RLE2 | 14.17 |
| | | RLE3 | 15.88 |
| | | RLE4 | 16.19 |
| | | RLE5 | 17.43 |
| | | RLE6 | 16.46 |
| | | RLE7 | 15.09 |
| | | RLE8 | 14.61 |
| | Ligature tongue | RLE1 | 0.00 |
| | | RLE2 | 0.00 |
| | | RLE3 | 0.00 |
| | | RLE4 | 0.00 |
| | | RLE5 | 0.00 |
| | | RLE6 | 0.00 |
| | | RLE7 | 0.00 |
| | | RLE8 | 0.00 |
| | Remove ligature | RLE1 | 14.98 |
| | | RLE2 | 15.32 |
| | | RLE3 | 15.00 |
| | | RLE4 | 12.25 |
| | | RLE5 | 15.11 |
| | | RLE6 | 16.64 |
| | | RLE7 | 15.81 |
| | | RLE8 | 15.89 |
| *Uropsilus gracilis* | None (before experiment) | UGR2 | 13.25 |
| | | UGR5 | 11.37 |
| | | UGR6 | 11.64 |
| | | UGR10 | 12.43 |
| | | UGR15 | 8.72 |
| | Ligature tongue | UGR2 | 0.00 |
| | | UGR5 | 0.00 |
| | | UGR6 | 0.00 |
| | | UGR10 | 0.00 |
| | | UGR15 | 0.00 |
| | Remove ligature | UGR2 | 10.13 |
| | | UGR5 | 7.97 |
| | | UGR6 | 8.12 |
| | | UGR10 | 7.93 |
| | | UGR15 | 10.11 |
| *Typhlomys daloushanensis* | None (before experiment) | TDA1 | 34.91 |
| | | TDA2 | 32.30 |
| | | TDA3 | 34.03 |
| | | TDA4 | 29.11 |
| | | TDA5 | 33.21 |
| | Ligature tongue | TDA1 | 33.42 |
| | | TDA2 | 29.98 |
| | | TDA3 | 29.30 |
| | | TDA4 | 28.01 |
| | | TDA5 | 31.30 |
| | Remove ligature | TDA1 | 35.71 |
| | | TDA2 | 30.31 |
| | | TDA3 | 29.27 |
| | | TDA4 | 33.63 |
| | | TDA5 | 29.52 |

**Table S5. Performance of *Uropsilus* gracilis with tongue ligature on the disc-platform apparatus.**

| Experiment treatment | Test apparatus | Sample # | Percentage of exploration time in monitored sector (%) | # of ultrasonic emissions per sec in monitored sector |
|---|---|---|---|---|
| Tongue ligation | Disc-platform | UGR2 | 12.27 ± 2.16 | 0.00 |
| | | UGR5 | 14.25 ± 1.18 | 0.00 |
| | | UGR6 | 13.60 ± 1.38 | 0.00 |
| | | UGR10 | 13.50 ± 2.00 | 0.00 |
| | | UGR15 | 14.53 ± 1.37 | 0.00 |
| None（removeligation） | Disc-platform | UGR2 | 31.48 ± 1.40 | 0.00 |
| | | UGR5 | 29.51 ± 3.16 | 0.00 |
| | | UGR6 | 24.50 ± 1.40 | 0.00 |
| | | UGR10 | 32.56 ± 0.50 | 0.00 |
| | | UGR15 | 27.55 ± 1.27 | 0.00 |

**Table S6. Echolocating species in mammals.**

| | | Total mammals | Echolocating mammals | Proportion |
|---|---|---|---|---|
| Taxa | Species | 6497 | 1364 | 0.210 |
| | Genera | 1314 | 235 | 0.179 |
| | Families | 167 | 34 | 0.204 |
| | Orders | 27 | 5 | 0.185 |

Echolocation mammals species

| | | Bats | Toothed whales | Shrews | Tenrecs | Soft-furred tree mice |
|---|---|---|---|---|---|---|
| Taxa | Species | 1266 | 82 | 7 | 3 | 6 |
| | Genera | 191 | 36 | 4 | 3 | 1 |
| | Families | 21 | 10 | 1 | 1 | 1 |
| | Orders | 1 | 1 | 1 | 1 | 1 |
